# *TP53* mutation at codon 179 metabolically reprograms cancer cells to promote invasion

**DOI:** 10.1038/s41417-026-01045-4

**Published:** 2026-06-05

**Authors:** Debina Sarkar, Gaurav C. Gyanwali, Jaishree Patel, Polina Shevchuk, Aaron Jeffs, Adele G. Woolley, Selena Wang, Maryam Sardari, Apeksha Bhandarkar, Halima Siddiqui, Magdalena Ratajska, Makhdoom Sarwar, Srinivasaraghavan Kannan, Chandra S. Verma, Catherine J. Drummond, Cushla McKinney, Jisha Antony, Sally P. McCormick, Antony Braithwaite, Sunali Mehta

**Affiliations:** 1 Department of Pathology and Molecular Medicine, University of Otago, Dunedin, New Zealand; 2 Maurice Wilkins Centre for Biodiscovery, Auckland, New Zealand; 3 Department of Biochemistry, University of Otago, Dunedin, New Zealand; 4 Division of Pathology and Neuropathology, Medical University of Gdansk, Gdansk, Poland; 5 Department of Obstetrics and Gynaecology, University of Otago, Christchurch, New Zealand; 6 Bioinformatics Institute, Agency for Science, Technology and Research (A*STAR), Singapore, Singapore; 7 Department of Biological Sciences, National University of Singapore, Singapore, Singapore; 8 School of Biological Sciences, Nanyang Technological University, Singapore, Singapore

**Keywords:** Cancer models, Cancer metabolism, Cell biology

## Abstract

Mutations in the tumour suppressor, *TP53*, are prevalent in human cancers yet their functional implications remain unclear. Through comprehensive pan-cancer database analysis, we identified H179 mutations in *TP53* as significantly associated with poor disease-free survival across multiple cancer types. Functional studies in lung, ovarian, and prostate cancer cells over-expressing the p53^H179R/Y^ mutants revealed that these mutants are not only defective in tumour-suppressive functions but also actively promote tumour progression. These mutants drive metabolic reprogramming by increasing neutral lipid levels through de novo fatty acid synthesis (p53^H179R/Y^) and fatty acid uptake (p53^H179Y^), accompanied by increased lipid droplets and APOE expression, collectively promoting enhanced invasiveness relative to p53-null cells. Our findings demonstrate that *TP53* missense mutations are not functionally equivalent to *TP53* loss. Notably, H179 mutations act as oncogenic drivers, and our data highlight APOE and lipid metabolism as potential therapeutic targets, underscoring the clinical relevance of precise *TP53* mutation characterisation.

## Introduction

The p53 tumour suppressor protein is a transcription factor that plays a central role in maintaining the integrity of the genome by promoting repair, senescence, or apoptosis of DNA-damaged cells [1]. Loss of p53 function is often a precursor for tumour initiation and progression, which has been demonstrated by increased cancer risk in Li-Fraumeni syndrome patients with germline pathogenic variants, and p53 knockout mouse models [2, 3]. Unlike other tumour suppressor genes where loss of function occurs due to loss of heterozygosity, frameshift, or nonsense mutations, the majority of pathogenic *TP53* mutations (~65%) are single nucleotide missense variants (Supplementary Fig. 1A). Missense mutations at codons 175, 220, 245, 248, 249, 273, and 282 occur at a higher-than-expected frequency and are referred to as hotspots; however, they constitute of only one-third of reported missense p53 mutations [4]. Numerous studies have deepened our understanding of the structure and function of p53, yet they have not resulted in clinical applications. Lack of clinical utility can be attributed to the fact that not all p53 mutations are the same in terms of how they affect patient outcomes, p53 function, biological pathways, and response to therapy [5–11]. This is further compounded by the fact that there is little evidence on how the remaining two-thirds of the reported missense p53 mutations influence p53 function, biological processes, and, in turn, response to therapy and patient outcome. Emerging evidence shows that mutant p53 not only disrupts tumour-suppressive functions but actively promotes oncogenic metabolic reprogramming by enhancing glycolysis through upregulation of GLUT1 and HK2 [12], stimulating lipid synthesis via SREBP activation [13], and inducing redox imbalance through altered NADPH and glutathione metabolism [14–16]. These changes fuel tumour progression and contribute to more aggressive, therapy-resistant cancers.

Zinc ion plays an important role in promoting p53 protein folding, which is essential for tetramer formation and DNA binding in order for p53 to function as a transcription factor. Mutations that decrease p53 stability or zinc-binding affinity can promote tumour progression by both the loss of canonical p53 function and gain of alternative functions. One such mutation is R175H, which is known to reduce metal-binding affinity [17] and promote gain of function such as inducing genetic instability [18], promoting tumour migration, invasion and metastasis [19–23], conferring cancer cell stemness [24–26] and drug resistance [27–29]. Amino acids at positions 176, 179, 238, and 242 play an important role in the tetrahedral coordination of a single zinc ion [30]. Results from thermodynamic modelling have identified that, similar to R175H, mutations at codons 179, 176, and 242 are likely to exhibit decreased zinc ion affinity [30]. Interestingly, our pan-cancer analysis has identified that mutations at H179 are reported at a similar frequency to the hotspot mutations at codons 245, 220, and 282 (Supplementary Fig. 1B). However, there is a paucity in the literature regarding the impact H179 mutations have on p53 function, cancer cell biology, and its association with patient outcome. Moreover, *TP53* mutation status is often still interpreted clinically as equivalent to *TP53* loss, an assumption that may overlook the distinct biological and clinical consequences of specific missense mutations such as H179.

In this study, we have capitalised on the integrated pan-cancer dataset from The Cancer Genome Atlas [31] (TCGA) and the MSK-IMPACT [32] datasets to identify that cancers with H179 *TP53* mutations are associated with poor patient outcomes. Furthermore, using models of lung, ovarian, and prostate cancer cells, we show that p53^H179R/Y^ mutants lose canonical p53 function and metabolically reprogramme cells by increasing lipid levels either via de novo synthesis and/or fatty acid uptake, along with an increase in lipid droplets and expression of apolipoprotein E (APOE), and stabilisation of vimentin (VIM) fibres. This metabolic reprogramming drives a more invasive phenotype, with mutant cells exhibiting greater invasiveness in 3D collagen matrices compared to *TP53*-null cells.

## Results

### H179 *TP53* mutations are present in multiple tumour types and are associated with poor disease-free survival

**Fig. 1:**
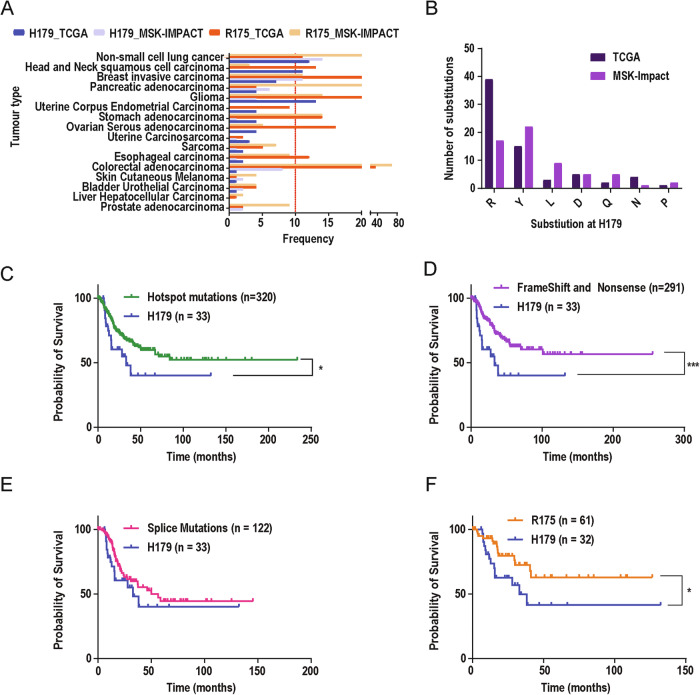
Mutations at codon 179 are present across multiple tumour types and associated with poor disease-free survival. **A** Prevalence of mutations at codons 179 and 175 in multiple tumour types. **B** Frequency of amino acid substitutions reported at codon 179 in multiple tumour types. Number of H179 *TP53* mutant tumours from TCGA: *n* = 69 and MSK-IMPACT: *n* = 61. **A**, **B** Data from TCGA and MSK-IMPACT is available via cBioPortal [73]. **C**–**F** Kaplan–Meier plots show disease-free survival (DFS) of patients from matched tumour types in the TCGA pan-cancer dataset (via cBioPortal), stratified by tumours harbouring H179 *TP53* mutations versus different *TP53* mutation categories. **C** H179 mutations were compared to hotspot mutations at codons 175, 220, 245, 248, 273, and 282 (H179: *n* = 33, events = 15; hotspot: *n* = 320, events = 93; *p* = 0.025). **D** Frameshift and nonsense mutations (H179: *n* = 33, events = 15; frameshift/nonsense: *n* = 291, events = 66; *p* = 0.0008). **E** Splice mutations (H179: *n* = 33, events = 15; splice: *n* = 122, events = 42; *p* = 0.2). **F** *TP53* mutations at codon 175 (H179: *n* = 32, events = 14; R175: *n* = 61, events = 14; 0.032). Statistical significance was determined using the log-rank test (*p < 0.05, **p < 0.01, ***p < 0.005, ****p < 0.0001).

Analysis of pan-cancer datasets from TCGA and MSK-IMPACT indicated that, similar to *TP53* R175 mutations, H179 mutations occur across multiple tumour types (Fig. 1A). H179 mutations are most frequent in non-small cell lung cancer in both datasets, although the differences compared with other tumour types are modest. By contrast, R175 mutations show higher prevalence in colorectal and several other cancer types (Fig. 1A). We also identified that single nucleotide changes at codon 179 results in a Histidine being mutated to an Arginine (R), Tyrosine (Y), Glutamine (Q), Proline (P), Asparagine (N), Leucine (L) and Aspartic acid (D) of which H179R and H179Y changes are more frequent in both datasets (Fig. 1B). To test the association of H179 *TP53* mutations with patient outcome, we conducted a matched tumour type disease-free survival study of H179 *TP53* mutations versus hotspot, frameshift, nonsense, and splice mutations using the pan-cancer dataset from TCGA. Our analyses showed that presence of H179 *TP53* mutations was significantly associated with poor disease-free survival compared to hotspot mutations (*p* = 0.0251, Fig. 1C). However, there was no significant difference between hotspot mutations and mutations present at codons 158, 193, 241, 249, 278 and 280 (Supplementary Fig. 1C). The presence of H179 *TP53* mutations was also associated with significantly poor disease-free survival compared to frameshift and nonsense mutations (*p* = 0.008, Fig. 1D) but not splice mutations (*p* = 0.2, Fig. 1E). Moreover, H179 *TP53* mutations were also associated with significantly worse disease-free survival compared to *TP53* mutations at codon R175 (*p* = 0.032, Fig. 1F). Taken together, these data suggest that mutations at codon 179 are present in multiple tumour types and may lead to loss of p53 function and alter tumour biology resulting in poor patient outcome.

### p53^H179R^ and p53^H179Y^ mutants are defective in wild-type p53 function

To test the effect of two common substitutions at codon 179 (H179R and H179Y) on p53 function identified from our bioinformatic analysis (Fig. 1B), we used site-directed mutagenesis to create pCMV-TP53^H179R^, pCMV-TP53^H179Y^, pCMV-TP53^R175H^ and pCMV-Vector (Vo – p53 null) from the pCMV-TP53^WT^ (wild-type-WT) plasmid (Supplementary Fig. 2A). We then transiently transfected p53 null H1299 lung adenocarcinoma cells and confirmed the expression of p53^WT^, p53^R175H^, p53^H179R^ and p53^H179Y^ mutants using western blot (Supplementary Fig. 2B).

**Fig. 2:**
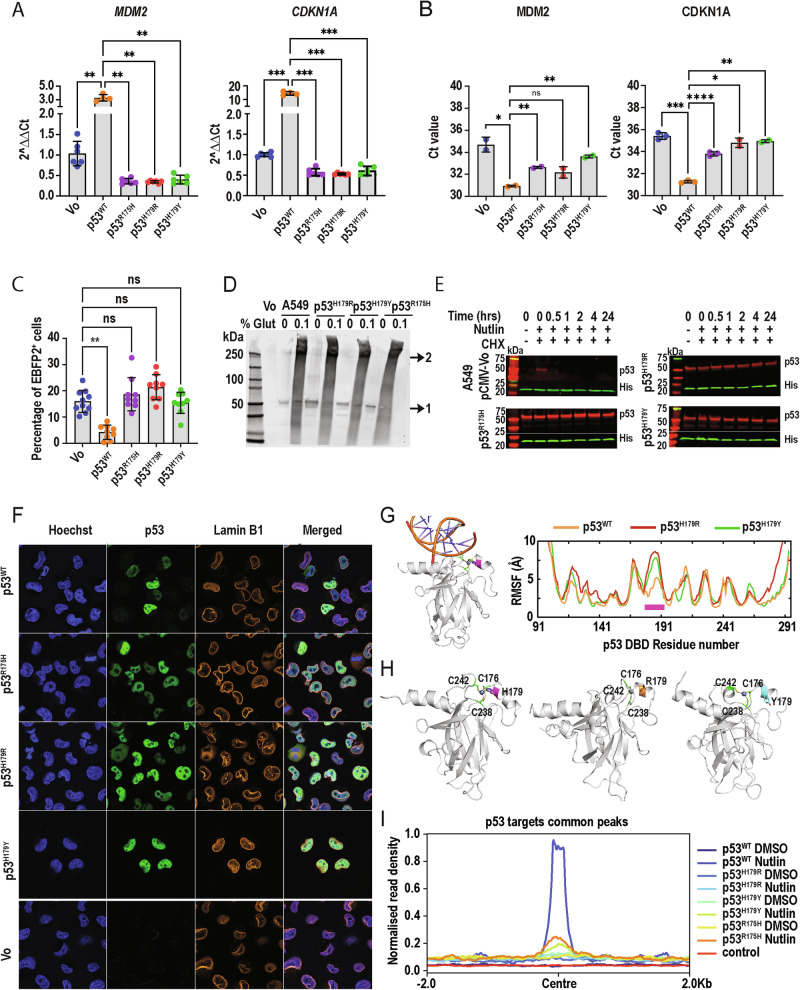
p53^H179R^ and p53^H179Y^ mutants are defective for p53 function, were able to form tetramers and localise to the nucleus. **A** RT-qPCR showing expression of *MDM2* and *CDKN1A* in H1299 cells 24 h post-transfection with 600 ng of plasmid expressing Vo or p53^WT^, p53^R175H^, p53^H179R^ and p53^H179Y^. **B** qPCR showing enrichment of *CDKN1A* and *MDM2* promoters when co-precipitated with anti-p53 antibody (DO-1) from H1299 cells 24 h post-transfection with 600 ng of plasmid expressing Vo or p53^WT^, p53^R175H^, p53^H179R^ and p53^H179Y^. **C** Quantification of EBFP2 positive cells 72 h post co-transfection of H1299 cells co-transfected with 600 ng of plasmids expressing either Vo or p53^WT^, p53^R175H^, p53^H179R^ and p53^H179Y^ along with 60 ng of pCMV-EBFP2 plasmids. **A**–**C** Significance was determined using Kruskal-Wallis and Welch tests and *p* < 0.05 was considered significant. * - *p* < 0.05, ** - *p* < 0.01, *** < 0.005, **** < 0.0001. **D** Western blot using anti-p53 antibody (DO-1) on either untreated or crosslinked (0.1% of glutaraldehyde) protein lysates from A549 (wt-p53) or H1299 cells stably expressing Vo, p53^R175H^, p53^H179R^ and p53^H179Y^. Monomer: 53 kDa and Tetramer: 212 kDa. **E** Western blot using anti-p53 antibody (DO-1) on protein lysate from either A549 cells stably expressing pCMV-Vector (Vo) or H1299 cells stably expressing p53^R175H^, p53^H179R^ and p53^H179Y^. Protein was collected from cells were treated with 10 μM nutlin-3A for 4 h followed by 50 μg/mL treatment with Cycloheximide for 0, 30 min, 1 h, 2 h, 4 h and 24 h. Anti-histone H3 antibody (EPR17785) was used to blot Histone-H3 (~15 kDa) as a loading control. **F** Representative images showing localisation of p53 in H1299 transiently transfected with 600 ng of plasmid expressing p53^WT^cells or stably expressing p53^R175H^, p53^H179R^ and p53^H179Y^ mutant protein p53 was stained with anti-p53 D0-1 antibody (1:1000, green), Lamin B1 was used as nuclear membrane marker which was stained with anti- Lamin B1 antibody (1:1000, red) and nuclei were stained with Hoechst 33258 (1:500, blue). **G** Left panel: structure of p53DBD^WT^ – DNA complex (pdb: 2AHI). DNA (orange) and p53 DBD^WT^ (grey) is shown as cartoon with the zinc (grey) shown as sphere and residues (C176, C238, C242 and H179) coordinating with zinc are highlighted in sticks (green and magenta). Right Panel: Average fluctuations of conformations of p53 DBD^WT^ and p53 DBD^MUT^ sampled during MD simulations. The zinc binding region that exhibits the increased fluctuations in the mutants are highlighted (magenta box). **H** MD Snapshot of p53 DBD (left: WT, middle: H179R and right H179Y) highlighting the conformational changes around the zinc binding site. **I** Profile plots of overlapping CUT&RUN peaks (*n* = 266, FDR q value ≤ 0.01) for 473 p53 target genes shows diminished signal in Nutlin-treated mutant cells compared to p53^WT^ (A549).

To assess whether the p53^H179R^ and p53^H179Y^ mutants retain wild-type p53 function, we measured mRNA expression of canonical p53 target genes 24 h post-transfection. H1299 cells expressing p53^WT^ displayed significantly higher mRNA levels of *CDKN1A* and *MDM2* (Fig. 2A), as well as *PIG3* (Supplementary Fig. 2C), compared to cells transfected with vector control (Vo) or the p53^R175H^, p53^H179R^, and p53^H179Y^ mutants. To determine whether reduced gene expression was associated with impaired DNA binding, promoter occupancy was performed for *CDKN1A* and *MDM2*. This revealed reduced promoter occupancy of Vo, p53^R175H^, p53^H179R^, and p53^H179Y^ compared with p53^WT^ at both loci (Fig. 2B), consistent with defective transcriptional activation by these mutant p53 proteins.

In parallel, we assessed the ability of the H179 mutants to suppress cell growth, by transiently co-transfecting H1299 cells with either Vo, pCMV-TP53^WT^, pCMV-TP53^R175H^, pCMV-TP53^H179R^, pCMV-TP53^H179Y^ along with pCMV-EBFP2 plasmid. We then calculated the percentage of pCMV-EBFP2 positive cells 72 h post-transfection as a marker of surviving transfected cells. As expected, significantly lower number of cells expressing p53^WT^ protein survived compared to cells expressing Vo or p53^R175H^, p53^H179R^ and p53^H179Y^ mutants (Supplementary Fig. 2D and Fig. 2C). Taken together these experiments provide evidence that p53^H179R^ and p53^H179Y^ mutants are defective in performing p53^WT^ function.

To explain the lack of p53^WT^ function by the p53^H179R^ and p53^H179Y^ mutants, we tested their ability to form tetramers, stability, and cellular localisation. To do this, we first created isogenic H1299 cells stably expressing either no p53 (Vo), p53^R175H^, p53^H179R^ or p53^H179Y^ mutants and confirmed the expression of these proteins using western blot (Supplementary Fig. 2E). In parallel, we also created *TP53*^WT^, A549 cells (functional p53) stably expressing pCMV-Vector (Vo) as a control. Since p53^WT^ must form a tetramer to function as a transcription factor [33], the loss of p53 function by p53^H179R^ and p53^H179Y^ mutants might be explained by their inability to form a tetramer. To test this, we performed western blot using either crosslinked (0.1% glutaraldehyde) or untreated protein lysate collected from either A549 (p53^WT^) or H1299 cells expressing Vo, p53^R175H^, p53^H179R^ and p53^H179Y^. Interestingly all the mutants p53^R175H^, p53^H179R^ and p53^H179Y^ retained their ability to form tetramers like the p53^WT^ protein (Fig. 2D). Next, we tested the stability of p53^H179R^ and p53^H179Y^ proteins, we treated either the A549 Vo or H1299 cells expressing p53^R175H^, p53^H179R^ and p53^H179Y^ with nutlin-3A followed by cycloheximide. As expected, nutlin-3A stabilised the expression of p53^WT^ protein as observed in A549 Vo cells and had a half-life of ~30 min (Fig. 2E), whereas nutlin-3A had no effect on p53^R175H^ and this protein had a half-life of longer than 24 h (Fig. 2E). Similarly, nutlin-3A had no effect on either the p53^H179R^ or p53^H179Y^ and these proteins also had a half-life greater than 24 h (Fig. 2E). Next, we determined the ability of the mutants to localise to the nucleus. Our results showed that all the mutants can localise to the nucleus like the p53^WT^ protein (Fig. 2F).

To further investigate structural consequences of H179 mutations, we performed molecular dynamics (MD) simulations of the p53^WT^ DNA-binding domain (DBD^WT^) and the p53^H179R^ and p53^H179Y^ mutants (DBD^H179R/Y^). H179 is located within a short α-helix and coordinates the zinc ion (Fig. 2G); substitution with R or Y disrupts this interaction, resulting in partial unfolding and reorientation of this helical segment and conformational changes in the surrounding region (Fig. 2H). Although both DBD^WT^ and DBD^H179R/Y^ remained globally stable, RMSD analysis showed ~3 Å for DBD^WT^ versus >5 Å for the mutants, indicating increased structural perturbation (Fig. 2G). The overall fold was largely preserved, but the mutant DBDs exhibited increased flexibility, particularly at the zinc-binding site and in loops L2 and L3 that interact with the DNA minor groove and are stabilised by zinc coordination (Fig. 2H).

To assess the functional consequences of these structural changes, CUT&RUN followed by sequencing was performed. Upon p53 stabilisation in A549 cells, p53^WT^ showed strong enrichment at known p53 target gene loci, whereas binding was reduced in p53^R175H^ and further diminished in p53^H179Y^. In contrast, p53^H179R^ and Vo cells showed little to no detectable binding (Fig. 2I).

Collectively, these results indicate that while the H179 mutants retain key structural features such as tetramerization, nuclear localisation, and overall fold, increased flexibility within the DNA-binding domain, impairs DNA binding, reduces transcription of canonical targets, and compromises tumour suppressor function. Notably, the phenotypes observed in p53^H179R^ and p53^H179Y^expressing cells consistently exceeded those seen in *TP53* null cells, demonstrating that these mutants are not simply loss-of-function variants but actively impose distinct oncogenic programmes.

### p53^H179R^ and p53^H179Y^ mutants is associated with altered lipid metabolism and transport and epithelial to mesenchymal transition

**Fig. 3:**
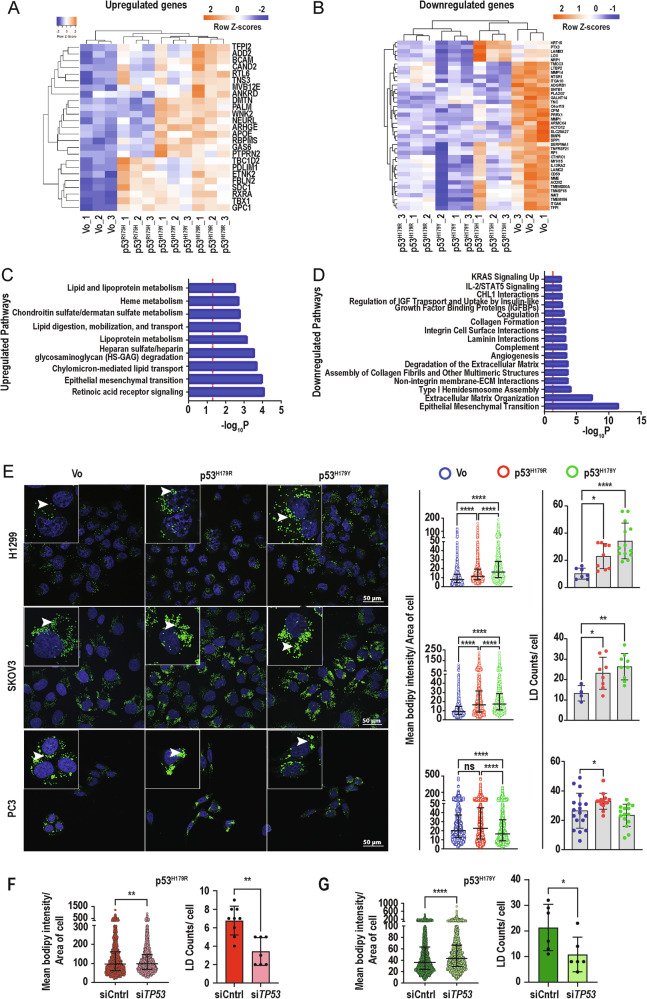
RNA-sequencing identifies lipid and EMT programmes associated with p53^H179^ mutants, confirmed by increased lipid accumulation. Heatmap showing z-scores of common differentially **A** Upregulated and **B** Downregulated mRNA transcripts in p53^H179R^ and p53^H179Y^ mutants compared to Vo and p53^R175H^ mutants in H1299 cells. Log fold change >0.5, Adj. *p* < 0.01. Heatmap generated using heatmapper [105]. **C** Common upregulated (left pane) and downregulated (right panel) pathways and biological processes significantly enriched in H1299 cells stably expressing p53^H179R^ and p53^H179Y^ mutants compared to Vo and p53^R175H^ mutants. Data obtained from EnrichR [103], Adjusted *p* < 0.05. **D** mRNA expression of *APOE*, *GPC1*, *SDC1* and *RXRA* in tumours with either deleted *TP53* (*TP53*_Deep Deletion, *n* = 77), H179 mutations (*TP53_H179*, *n* = 49) or R175H mutations (*TP53_R175H*, *n* = 141). Bars ± error bars: mean ± SEM. Significance was determined using Kruskal-Wallis test and *p* < 0.05 was considered significant. Data was obtained from the pan-cancer dataset TCGA, cBioPortal [73]. **E** Representative images and quantitation of neutral lipids in H1299, SKOV3 and PC3 cells expressing p53^H179R^ and p53^H179Y^ mutant protein or Vo and stained for neutral lipid. Neutral lipid was stained with BODIPY™ 493/503 (1:1000 green) and nuclei were stained with Hoechst 33258 (2 μg/ mL, blue). Bars ± error bars: Mean ± SD (*n* = 3–5 fields and >200 cells). Representative images were acquired using a 60x oil-immersion objective and for quantitation, images were acquired using 20x objective and 60x oil-immersion objective. Quantitation of neutral lipids and lipid droplets in **F** p53^H179R^ and **G** p53^H179Y^, H1299 cells transiently transfected for 96 h with 20 nM control siRNA (siCntrl) or siRNA targeted *TP53* (si*TP53*). Significance was determined using Welch’s, Kruskal-Wallis and Mann–Whitney t-test and *p* < 0.05 was considered significant. * - *p* < 0.05, ** - *p* < 0.01, *** < 0.005, **** < 0.0001.

To determine the biological processes affected by p53^H179R^ and p53^H179Y^ mutants we carried out RNA sequencing on isogenic H1299 cells stably expressing either Vo or p53^R175H^, p53^H179R^ and p53^H179Y^ mutants. We identified upregulation of 78 genes by p53^H179R^ and 211 genes by p53^H179Y^, of these genes 26 were commonly upregulated by both p53^H179R^ and p53^H179Y^ mutants (Supplementary Fig. 3A and Fig. 3A). We also identified downregulation of 101 genes by p53^H179R^ and 169 genes by p53^H179Y^, of these 41 were commonly downregulated by both p53^H179R^ and p53^H179Y^ mutants (Supplementary Fig. 3B and Fig. 3B). These common upregulated genes were significantly enriched (q value < 0.05) for pathways involved either directly or indirectly in epithelial to mesenchymal transition (EMT) and lipid metabolism and transport (Fig. 3C, left panel). Interestingly the common downregulated genes were significantly enriched (q value < 0.05) for pathways involved in cell maintaining the attachment of epithelial cells to underlying basement membrane (Fig. 3D, right panel). The presence of EMT-related pathways among both up- and downregulated genes likely reflects context-dependent regulation where upregulated genes promote mesenchymal traits, while downregulated genes correspond to epithelial maintenance programmes, indicating that p53^H179R^ and p53^H179Y^ mutants modulate multiple aspects of EMT-related gene expression.

These transcriptional changes suggest that p53^H179R^ and p53^H179Y^ mutants may functionally influence both lipid metabolism and EMT-related processes, providing a rationale to examine whether these molecular programmes manifest as altered lipid accumulation and associated cellular phenotypes.

### Cancer cells expressing p53^H179R^ and p53^H179Y^ mutants have higher lipid content and increased number of lipid droplets compared to p53 null cells

Reprogramming of lipid metabolism either via altering rates of de novo synthesis, uptake or both are features exploited by cancer cells to promote cell survival and metastasis [34]. Our findings from RNA sequencing suggested an increase in lipid metabolism and transport, therefore, we wanted to test whether p53^H179R^ and p53^H179Y^ mutants increased lipid content in cancer cells. To do this, we stained the isogenic H1299 cells for neutral lipids with BODIPY™ 493/503. We found that H1299 cells expressing the p53^H179R^ and p53^H179Y^ mutants had elevated levels of neutral lipids compared to Vo cells (Fig. 3E, top panel), whereas no such increase was detected in cells expressing p53^WT^ (Supplementary Fig. 3C, D).

Excess lipid is commonly stored by cancer cells in specialised organelles known as lipid droplets, which are composed of the neutral lipid core [34, 35]. Lipid droplets are highly dynamic structures that serve as a rapid energy source for cancer cells and protect them from lipid overload and oxidative stress [34, 36]. Consistent with this we observed that the excess lipid found in H1299 cells expressing p53^H179R^ and p53^H179Y^ mutants was packaged in lipid droplets and the mutant cells showed significantly higher number of lipid droplets per cell compared to Vo (Fig. 3E, white arrows, top panel). In line with the absence of differences in total lipid levels between H1299 p53 null (Vo) and p53^WT^ cells, no significant change in lipid droplet number was observed in p53^WT^ expressing H1299 cells (Supplementary Fig. 3C–D).

To determine whether these findings extended beyond lung cancer cells, we generated isogenic ovarian (SKOV3) and prostate (PC3) cancer cell models expressing either no p53 (Vo) or the p53^H179R^ or p53^H179Y^ mutants (Supplementary Fig. 3E and 3F). Consistent with our findings in H1299 cells, SKOV3 cells expressing p53^H179R^ and p53^H179Y^ mutants had elevated levels of neutral lipids, and an increase in the number of lipid droplet numbers, compared to Vo cells (Fig. 3E, middle panel). Interestingly, PC3 cells expressing p53^H179R^ had similar levels of neutral lipids and a marginal increase in lipid droplets per cell compared to Vo cells (Fig. 3E, bottom panel). In contrast, PC3 cells expressing p53^H179Y^ mutants had reduced levels of neutral lipid and similar number of lipid droplets per cell compared to Vo cells (Fig. 3E, bottom panel). Taken together, these results show that in the presence of p53^H179R^ and p53^H179Y^ mutants, both neutral lipid levels and the number of lipid droplets are increased, with robust effects in lung and ovarian cancer cells but more variable effects in prostate cancer cells.

Next to test whether p53^H179R^ and p53^H179Y^ mutants directly regulate lipid accumulation, we transiently knocked down mutant p53 using either a control small interfering RNA (siRNA) or one targeting *TP53*, to knockdown p53^H179R^ and p53^H179Y^ (Supplementary Fig. 3G). These experiments were performed in the presence of serum (FBS), where extracellular lipid availability is not restricted. We then stained the p53^H179R^ and p53^H179Y^ cells treated with either control siRNA or siRNA targeting *TP53* for neutral lipids with BODIPY™ 493/503 (Supplementary Fig. 3H). Under these conditions, transient knockdown of p53^H179R^ and p53^H179Y^ resulted in a significant decrease in the number of lipid droplets per cell (Fig. 3F, G), despite an overall increase in neutral lipid levels. This increase in bulk lipid content is likely influenced by the continued availability of serum-derived lipids and the transient nature of p53 depletion, whereas the reduction in lipid droplet number suggests a role for mutant p53 in regulating lipid droplets under these conditions.

These observations prompted us to next investigate whether the increased lipid levels arise from enhanced de novo fatty acid synthesis, increased uptake of extracellular lipids, or a combination of both.

### p53^H179R^ increases de novo fatty acid synthesis while p53^H179Y^ also increases fatty acid uptake to reprogramme lipid metabolism in cancer cells

**Fig. 4:**
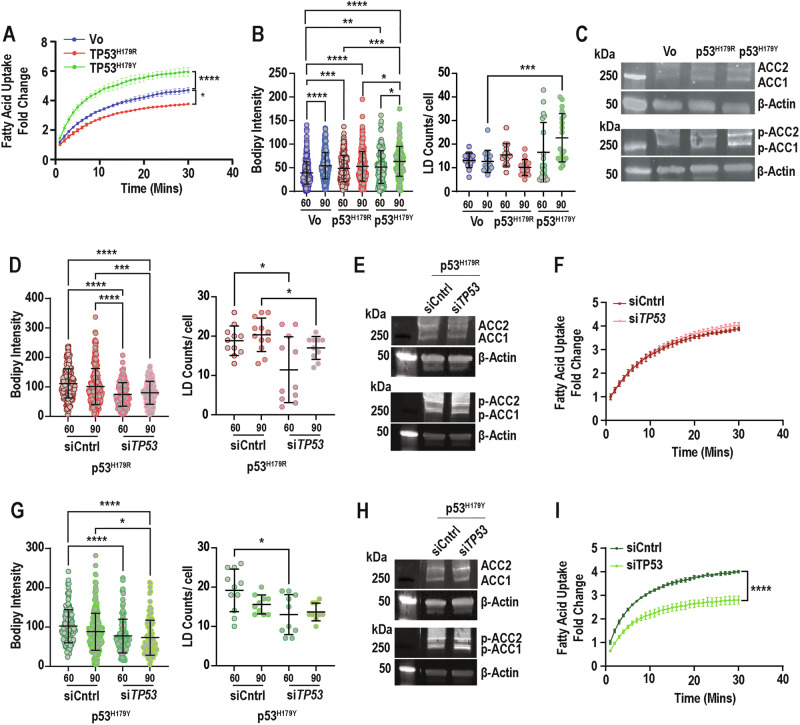
*TP53*^*H179R*^ promotes lipid accumulation through fatty acid synthesis, whereas *TP53*^*H179Y*^ does so through both synthesis and uptake. **A** Kinetics of fatty acid uptake in serum-starved H1299 cells expressing p53^H179R^ and p53^H179Y^ mutant protein or Vo. **B** Quantitation of neutral lipids (left panel) and lipid droplets (right panel) in H1299 cells expressing p53^H179R^ and p53^H179Y^ mutant protein or Vo after the cells were serum starved for 60 mins and 90 mins. **C** Western blot using anti-ACC antibody (3662, 1:1000) and anti-phosho-ACC, (3661, 1:1000) on protein lysate from either H1299 cells expressing p53^H179R^ and p53^H179Y^ mutant protein or Vo, 60 min post serum starvation (~265 kDa for ACC1/pACC1 and ~280 kDa for ACC2/pACC2). Anti-β-Actin (SP124, 1:5000) was used to blot β-Actin as a loading control (~45 kDa). **D** Quantitation of neutral lipids and lipid droplets in p53^H179R^ H1299 cells transiently transfected for 96 h with 20 nM control siRNA (siCntrl) or siRNA targeted *TP53* (si*TP53*). **E** Western blot using anti-ACC antibody (3662, 1:1000) and anti-phospho-ACC (3661, 1:1000) on protein lysate from p53^H179R^ H1299 cells transiently transfected for 96 h with 20 nM control siRNA (siCntrl) or siRNA targeted *TP53* (si*TP53*). **F** Kinetics of fatty acid uptake in H1299 cells expressing p53^H179R^ mutant protein which were transiently transfected for 72 h with 20 nM control siRNA (siCntrl) or siRNA targeted *TP53* (si*TP53*). **G** Quantitation of neutral lipids and lipid droplets in p53^H179Y^ H1299 cells transiently transfected for 96 h with 20 nM control siRNA (siCntrl) or siRNA targeted *TP53* (si*TP53*). **H** Western blot using anti-ACC antibody (3662, 1:1000) and anti-phospho-ACC (3661, 1:1000) on protein lysate from p53^H179Y^ H1299 cells transiently transfected for 96 h with 20 nM control siRNA (siCntrl) or siRNA targeted *TP53* (si*TP53*). **I** Kinetics of fatty acid uptake in H1299 cells expressing p53^H179Y^ mutant protein which were transiently transfected for 72 h with 20 nM control siRNA (siCntrl) or siRNA targeted *TP53* (si*TP53*). **B**, **D**, **G** Significance was determined using Welch’s, Kruskal-Wallis and Mann-Whitney t-test and *p* < 0.05 was considered significant. **A**, **F**, **I** Significance was determined using one-way ANOVA, Brown-Forsyth and Welch ANOVA tests and *p* < 0.05 was considered significant. * - *p* < 0.05 was considered significant. * - *p* < 0.05, ** - *p* < 0.01, **** < 0.0001 and ns non-significant.

Next, we tested whether the increase in lipid content was due to increased uptake of extracellular fatty acid which accounted for high lipid levels in the cells expressing p53^H179R^ and p53^H179Y^ mutants. To do this, we used a fluorescent long chain fatty acid analogue which acted as a substrate and examined the kinetics of fatty-acid uptake in 60 min serum-starved H1299 cells expressing p53^H179R^ and p53^H179Y^ mutants. Interestingly, we found that between p53^H179R^ and p53^H179Y^ mutant cells, only p53^H179Y^ expressing cells showed a significant increase in uptake of fatty acid compared to Vo cells (Fig. 4A).

Next we wanted to test, whether increase in lipid content was due to de novo fatty acid synthesis, we serum-starved the cells expressing the p53^H179R^ and p53^H179Y^ mutants for 60 min and 90 min following which we assessed the levels of neutral lipids by staining the cells with BODIPY™ 493/503. In parallel, we evaluated total acetyl-CoA carboxylase isoforms (ACC1 and ACC2) and their inhibitory phosphorylated forms (pACC1 and pACC2), which are key enzymes catalysing the rate-limiting step of de novo fatty acid synthesis [37]. We found that cells expressing p53^H179R^ showed increased lipid content at 60 min and maintained these levels at 90 min post serum starvation compared to the Vo cells at 60 min (Fig. 4B and Supplementary Fig. 4A), while the lipid content in cells expressing p53^H179Y^ mutants steadily increased from 60 min to 90 min compared to the Vo cells at 60 min (Fig. 4B and Supplementary Fig. 4A). Consistent with these changes, there was a trend toward increased lipid droplets in p53^H179R^ cells at 60 min; however, droplet numbers decreased by 90 min post-starvation compared to Vo cells (Fig. 4B and Supplementary Fig. 4A). Conversely, p53^H179Y^-expressing cells showed a trend toward increased lipid droplet counts at 60 min, which became significantly higher by 90 min post-starvation compared to Vo cells (Fig. 4B and Supplementary Fig. 4A). Both mutants exhibited higher total ACC1 and ACC2 protein levels 60 min post starvation (Fig. 4C), indicating increased potential for fatty acid synthesis. Although pACC1 and pACC2 levels were also elevated 60 min post starvation (Fig. 4C), which normally inhibits ACC activity, the overall increase in ACC abundance likely contributes to the observed lipid accumulation.

To determine the contribution of p53^H179R^ and p53^H179Y^ to lipid accumulation, we transiently transfected H1299 cells expressing each mutant with either control siRNA or *TP53* targeting siRNA. Seventy-two hours post-transfection, cells were serum-starved for 60 and 90 min. Using BODIPY™ 493/503 staining, we measured neutral lipid levels, and lipid droplet counts were quantified. In parallel, total levels of ACC1 and ACC2, as well as their inhibitory phosphorylated forms (pACC1 and pACC2), were assessed 60 min post starvation. Additionally, extracellular fatty acid uptake was evaluated using a fluorescent long-chain fatty acid analogue in a kinetic assay, post 60 min starvation, 72 h post transfection.

Knockdown of p53^H179R^ (Supplementary Fig. 4B) significantly reduced neutral lipid levels at both 60 min and 90 min post-serum starvation, and lipid droplet counts significantly decreased at both time points (Fig. 4D and Supplementary Fig. 4C). At the protein level, ACC2 expression decreased following p53^H179R^ depletion, while ACC1 and pACC1/pACC2 levels remained unchanged. Extracellular fatty acid uptake was not affected by p53^H179R^ knockdown (Fig. 4F). These results indicate that p53^H179R^ promotes lipid accumulation predominantly through enhanced de novo fatty acid synthesis, associated with increased ACC2 expression, and is reflected by elevated neutral lipid levels and lipid droplet abundance.

In p53^H179Y^ expressing cells, *TP53* knockdown (Supplementary Fig. 4B) significantly decreased neutral lipid levels at both 60 and 90 min (Fig. 4G and Supplementary Fig. 4D). Lipid droplet counts were significantly reduced at 60 min and trended lower at 90 min (Fig. 4G). ACC1 expression remained unchanged, while inhibitory pACC1 increased; ACC2 showed a marginal increase, with no change in pACC2 (Fig. 4H). Importantly, extracellular fatty acid uptake was significantly reduced upon p53^H179Y^ depletion (Fig. 4I), consistent with the observation that only p53^H179Y^ expressing cells exhibited increased fatty acid uptake compared to Vo controls (Fig. 4A). These findings indicate that p53^H179Y^ promotes lipid accumulation through a combination of de novo fatty acid synthesis and enhanced extracellular fatty acid uptake, and that knockdown reduces neutral lipid levels, lipid droplet abundance, and fatty acid uptake. The increase in pACC1 upon knockdown is observed alongside the reduction in lipid levels, suggesting a potential association with decreased fatty acid synthesis.

Together, these findings demonstrate that the p53^H179R^ and p53^H179Y^ mutants promote lipid metabolic reprogramming suggested by our RNA-sequencing analysis, with p53^H179R^ primarily enhancing de novo fatty acid synthesis and p53^H179Y^ promoting both synthesis and uptake, thereby contributing to the elevated lipid levels and lipid droplet accumulation observed in H179R/Y mutant p53 expressing cells.

### p53^H179R^ and p53^H179Y^ mutants upregulate apolipoprotein E (APOE) expression linking lipid metabolism to cytoskeletal remodelling

Building on these observations, we returned to our RNA-sequencing dataset to explore potential mediators of the metabolic changes driven by p53^H179R^ and p53^H179Y^. Among the common upregulated genes, *GPC1*, *SDC1*, *RXRA*, *PDLIM1*, *DMTN*, *PTPRN2*, and *APOE* were implicated in multiple enriched pathways, suggesting that these factors may contribute to the effects of the mutants on lipid metabolism and EMT.

**Fig. 5:**
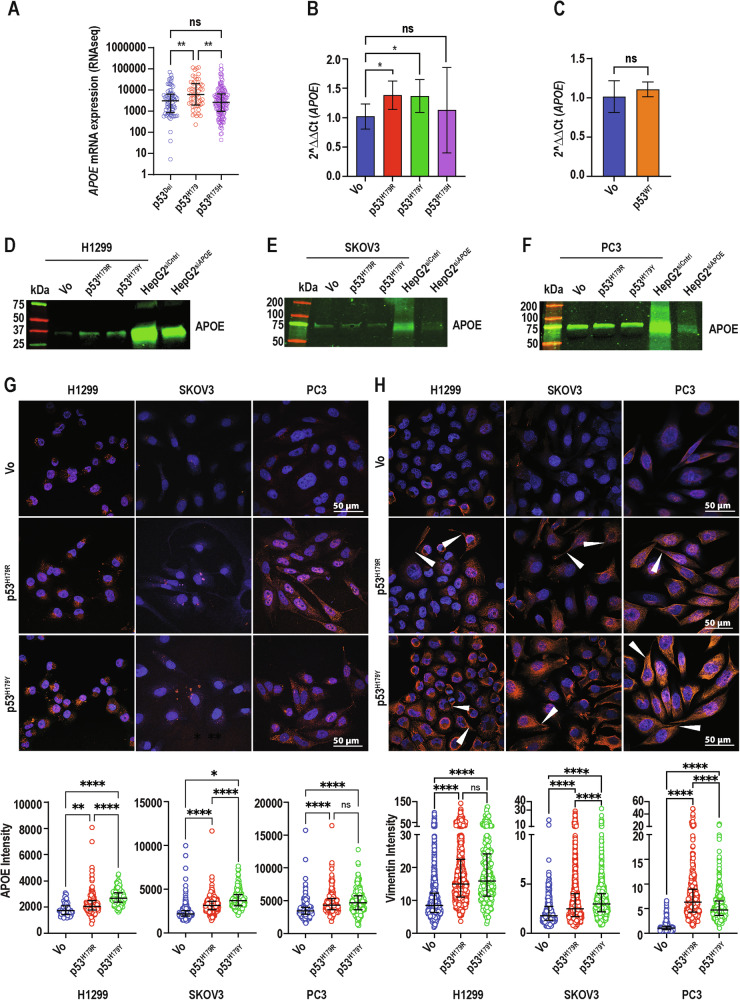
APOE is upregulated in *TP53*^*H179*^ mutant cells and is associated with increased protein levels and stabilisation of Vimentin. **A** Expression of *APOE* mRNA in tumours with either deleted *TP53* (*TP53*_Deep Deletion, *n* = 77), H179 mutations (*TP53_H179*, *n* = 49) or R175H mutations (*TP53_R175H*, *n* = 141). Bars ± error bars: mean ± SEM. Significance was determined using Kruskal-Wallis test and *p* value < 0.05 was considered significant. Data was obtained from the pan-cancer dataset TCGA, cBioPortal (PMID: 22588877). **B** Expression of *APOE* mRNA measured using RT-qPCR in H1299 cells expressing either Vo or p53^H179R^, p53^H179Y^ and p53^R175H^ mutants. **C** Expression of *APOE* mRNA measured using RT-qPCR in H1299 cells transiently transfected with either Vo or p53^WT^. **B**, **C** Bars ± error bars: mean ± SEM. **A**–**C** Significance was determined using Kruskal Wallis and Welch’s tests and *p* < 0.05 was considered significant. * - *p* < 0.05, ** - *p* < 0.01 and ns – non-significant. **D**–**F** Western blot using anti-APOE antibody (EPR19392, 1:1000) from media of Vo, p53^H179R^ and p53^H179Y^ cells cultured for 96 h (~34 kDa). **D** H1299, **E** SKOV3 and **F** PC3. Total protein stained with Ponceau S was used as loading control (Supplementary Fig. 6A–C). **G** Representative images and quantitation of intracellular APOE levels from H1299 (left panel), SKOV3 (middle panel) and PC3 (right panel) stably expressing Vo or p53^H179R^ and p53^H179Y^ mutants. APOE was stained with anti-APOE antibody (1:250, red) and nuclei were stained with Hoechst 33258 (2 μg/mL, blue). **H** Representative images and quantitation of Vimentin levels from H1299 (left panel), SKOV3 (middle panel) and PC3 (right panel) stably expressing Vo or p53^H179R^ and p53^H179Y^ mutants. VIM was stained with anti-VIM antibody (1:300, red) and nuclei were stained with Hoechst 33258 (1:500, blue). **G**, **H** Bars ± error bars: Mean ± SD (*n* = 3–5 fields and >200 cells). Representative images were acquired using a 60x oil-immersion objective and for quantitation, images were acquired using 20x objective. Significance was determined using Kruskal Wallis t-test and *p* < 0.05 was considered significant. * - *p* < 0.05, **** - *p* < 0.0001 and ns non-significant.

To assess these candidates in patient samples, we analysed RNA-sequencing data from the TCGA pan-cancer dataset, comparing tumours with *TP53*^*H179*^ mutations to those with *TP53* deletion (*TP53*^*del*^) or the *TP53*^*R175H*^ mutation. This analysis revealed that *APOE* and *GPC1* mRNA were significantly higher in *TP53*^*H179*^ tumours, whereas *SDC1* and *PDLIM1* were elevated in tumours with either *TP53*^*H179*^ or *TP53*^*R175H*^ mutations. In contrast, *PTPRN2* mRNA was decreased in *TP53*^*H179*^ tumours, and *RXRA* and *DMTN* expression did not differ from tumours with *TP53* deletion (Fig. 5A and Supplementary Fig. 5A).

To determine whether these findings were recapitulated in our cell models, we performed RT-qPCR in H1299 cells expressing p53^H179R^, p53^H179Y^, p53^R175H^, or Vo controls. *APOE* mRNA was significantly upregulated in p53^H179R/Y^ mutant but not in p53^R175H^ cells compared to Vo (Fig. 5B). *SDC1*, *PDLIM1* mRNA were significantly increased only in p53^H179R^ cells, while *PTPRN2* mRNA was upregulated solely in p53^H179Y^ cells (Supplementary Fig. 5B). *GPC1* mRNA expression was not altered in any of the cell lines (Supplementary Fig. 5B). Based on these results, and given the well-established role of APOE in lipid metabolism and transport, where it is abundantly associated with lipids in the interstitial fluid, lymph, and plasma, and has been implicated in neurodegenerative diseases and cancer (reviewed in [38]), we focused on *APOE* for further investigation as a potential mediator of p53^H179R/Y^ driven changes.

First, we tested whether p53^WT^ can modulate *APOE* mRNA levels. To do this, we stabilised p53 in A549 (p53^WT^) cells using nutlin-3A [39] or transiently expressed p53^WT^ in H1299 cells. These experiments showed that p53^WT^ did not alter *APOE* mRNA levels, indicating that *APOE* upregulation is specific to the H179R/Y mutants (Fig. 5C and Supplementary 5C). Interestingly, although p53^WT^ occupies the *APOE* promoter, it does not activate transcription, whereas the H179R/Y mutants fail to bind these regions, leading to increased *APOE* expression in mutant-expressing cells (Supplementary Fig. 5D). These findings suggest that *APOE* transcription maybe blocked by p53^WT^ protein which is released by the inability of the mutants to bind to these regions, which is how the mutants may transcriptionally contribute to the regulation of *APOE* mRNA, which in turn may contribute to their effects on lipid metabolism, transport, and EMT-related processes.

Given that APOE is a secreted protein, we next measured APOE protein levels in the culture media of H1299 cells expressing p53^H179R^ and p53^H179Y^ mutants. Consistent, with the mRNA data, both p53^H179R^ and p53^H179Y^ expressing H1299 cells had increased APOE secreted in media compared to Vo (Fig. 5D and Supplementary Fig. 6A). In contrast, there was no change in the expression of secreted APOE levels in p53^H179R^ and p53^H179Y^ expressing SKOV3 and PC3 cells (Fig. 5E, F and Supplementary Fig. 6B, C).

An additional difference we noted was that, in H1299 cells, APOE was predominantly secreted as a monomer (~32 kDa), whereas in SKOV3 and PC3 cells the predominant species detected was an APOE dimer (~74 kDa). Notably, dimerised APOE secretion has been previously reported in astrocytes and in the human frontal cortex and hippocampus [40, 41] supporting the physiological relevance of the dimeric species observed here. In HepG2 cells, APOE was secreted as both monomeric and dimeric forms. Importantly, siRNA-mediated knockdown of APOE in HepG2 cells resulted in the loss of both bands, confirming that both species correspond to APOE (Fig. 5D–F).

In addition to its extracellular functions, intracellular APOE is known to modulate various cellular processes such as assembly and stability of the cytoskeleton, the integrity and function of the mitochondria and the morphology and function of the dendrites in neurodegenerative disease including Alzheimer’s disease (reviewed in [38]). To determine whether p53^H179R^ and p53^H179Y^ expressing cells had elevated intracellular levels of *APOE*, we stained the cells with anti-APOE and Hoechst 33258 and quantitated the levels of intracellular APOE using immunofluorescence. Our results showed that H1299, SKOV3 and PC3 cells expressing p53^H179R^ and p53^H179Y^ had significantly higher levels of intracellular APOE compared to Vo cells (Fig. 5G). In contrast H1299 cells transiently transfected with p53^WT^ had significantly lower levels of intracellular APOE protein compared to Vo cells (Supplementary Fig. 6D). Notably, APOE localisation was cell-type and mutant dependent. In p53^H179R^ H1299 and SKOV3 cells, APOE was found predominantly in the cytoplasm, while in p53^H179R^ PC3 cells APOE localised both in the nucleus and cytoplasm. On the other hand, in p53^H179Y^ H1299 and PC3 cells, APOE was found in the cytoplasm and nucleus, but only in the cytoplasm of p53^H179Y^ SKOV3 cells (Fig. 5G). This heterogeneity suggested that APOE may exert distinct intracellular functions downstream of mutant p53.

APOE has been reported to modulate cytoskeletal dynamics through interactions with intermediate filaments such as vimentin (VIM) [42], a key regulator of cell shape and EMT [43–47]. This raised the possibility that elevated APOE in p53^H179R/Y^ mutant cells may contribute not only to altered lipid metabolism but also to EMT-associated phenotypes via VIM modulation. To investigate this, we first examined whether *VIM* mRNA was altered. RNA-sequencing analysis showed no significant changes in *VIM* transcript levels in p53^H179R/Y^ mutants compared to Vo (Supplementary Fig. 6E), and neither p53^WT^ nor mutant proteins occupied the VIM promoter (Supplementary Fig. 6F), indicating that regulation is unlikely to be transcriptional.

We therefore assessed VIM protein levels and organisation by immunofluorescence [48]. H1299, SKOV3, and PC3 cells expressing p53^H179R^ or p53^H179Y^ exhibited significantly increased VIM protein levels compared to Vo controls, whereas transient expression of p53^WT^ in H1299 cells did not produce this effect (Fig. 5H and Supplementary Fig. 6G). Moreover, VIM filaments in mutant-expressing cells were reorganised into structures such as lamellipodia and filopodia [48], consistent with enhanced cytoskeletal dynamics, while Vo cells displayed a more diffuse VIM distribution (Fig. 5H, white arrows).

**Fig. 6:**
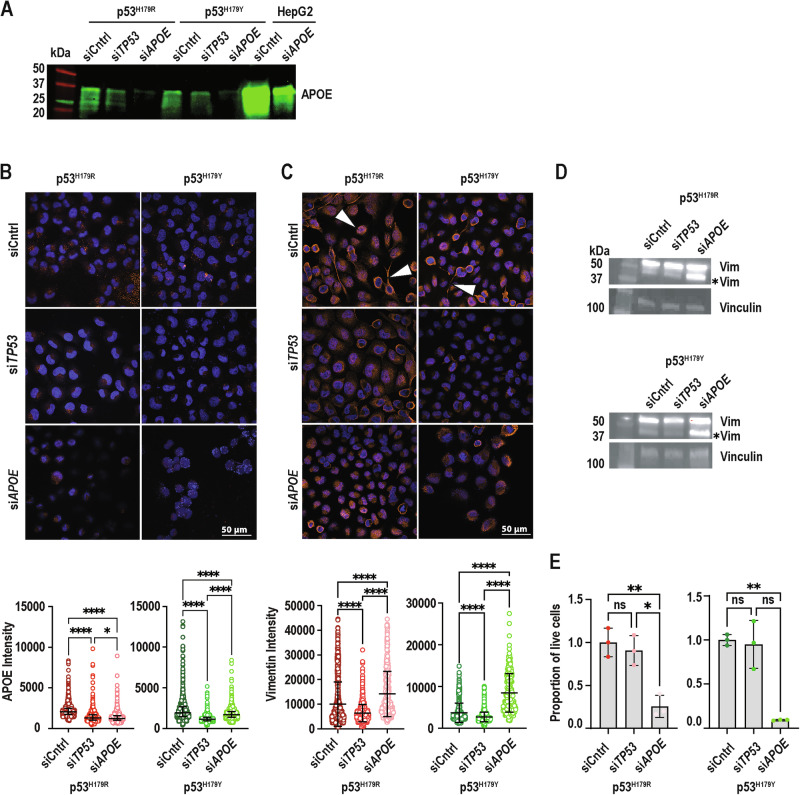
APOE is required for Vimentin stabilisation and cell viability in p53^H179R/Y^ expressing cells. **A** Western blot of conditioned media from H1299 cells expressing p53^H179R^ or p53^H179Y^ and treated for 96 h with control siRNA (siCntrl, 20 nM) or siRNAs targeting *TP53* (siTP53, 20 nM) or *APOE* (siAPOE, 5 nM), probed with anti-APOE antibody (EPR19392). Corresponding p53 immunoblots from cell lysates are shown in Supplementary Fig. 7A. β-actin (SP124) was used as a loading control for cell lysates, and total protein stained with Ponceau S served as a loading control for the media blot (Supplementary Fig. 7B). **B** Representative images and quantitation of intracellular APOE levels from H1299 p53^H179R^ and p53^H179Y^ expressing cells treated with either control siRNA (siCntrl) or those targeting either *TP53* (si*TP53*) or *APOE* (si*APOE*), cultured for 96 h. APOE was stained with anti-APOE antibody (1:250, red) and nuclei were stained with Hoechst 33258 (2 μg/mL, blue). Bars ± error bars: Mean ± SD (*n* = 3–5 fields and >200 cells). Representative images and quantitation of Vimentin levels from H1299 (top panel), SKOV3 (middle panel) and PC3 (bottom panel) stably expressing Vo or p53^H179R^ and p53^H179Y^ mutants. **C** Representative images and quantitation of VIM levels from H1299 p53^H179R^ and p53^H179Y^ expressing cells treated for 96 h with either control siRNA (siCntrl – 20 nM) or siRNA targeting *TP53* (si*TP53* – 20 nM). VIM was stained with anti-VIM antibody (1:300, red) and nuclei were stained with Hoechst 33258 (1:500, blue). Representative images were acquired using a 60x oil-immersion objective and for quantitation, images were acquired using 20x objective. Significance was determined using Kruskal Wallis t-test and *p* < 0.05 was considered significant. * - *p* < 0.05, **** - *p* < 0.0001 and ns non-significant. **D** Western blot using either anti-VIM antibody (EPR3776, 1**:**1000) on protein lysates from H1299 cells expressing p53^H179R^ and p53^H179Y^ and treated for 96 h with either control siRNA (siCntrl – 20 nM) or those targeting either *TP53* (si*TP53* – 20 nM) or *APOE* (si*APOE* – 5 nM). Vim: ~ 53 kDa, *Vim (C-terminus cleaved product): ~48 kDa. Anti-Vinculin antibody (VPA00888, 1:5000) was used to blot Vinculin (~124 kDa) as a loading control. **E** Proportion of live H1299 p53^H179R^ and p53^H179Y^ expressing cells treated with either control siRNA (siCntrl) or those targeting either *TP53* (si*TP53*) or *APOE* (si*APOE*), cultured for 96 h. Significance was determined using Kruskal Wallis t-test and *p* < 0.05 was considered significant. * - *p* < 0.05, **** - *p* < 0.0001 and ns non-significant.

To determine whether mutant p53 directly regulates APOE levels, we transiently knocked down p53^H179R^ or p53^H179Y^ in H1299 cells using siRNA. Efficient knockdown was confirmed by immunoblotting (Supplementary Fig. 7A). Depletion of either mutant resulted in a reduction in secreted APOE levels (Fig. 6A and Supplementary Fig. 7B), accompanied by a significant decrease in intracellular APOE as assessed by immunofluorescence (Fig. 6B). These data demonstrate that p53^H179R^ and p53^H179Y^ regulate both intracellular and secreted APOE.

Given the link between APOE and cytoskeletal regulation, we next examined whether APOE mediates VIM organisation downstream of mutant p53. Knockdown of p53^H179R^ or p53^H179Y^ (Supplementary Fig. 7A), resulted in reduced VIM levels and loss of organised VIM filament structures compared to control siRNA-treated cells (Fig. 6C). In contrast, APOE knockdown increased overall VIM levels but caused pronounced disruption of VIM fibre organisation (Fig. 6C). Notably, immunoblotting revealed the presence of a cleaved C-terminal VIM fragment exclusively in APOE depleted cells, which was not detected following control or mutant *TP53* knockdown (Fig. 6D). C-terminal VIM cleavage has previously been associated with cytoskeletal destabilisation and increased cell death [49]. Consistent with this, APOE depletion significantly reduced cell viability in both p53^H179R^ and p53^H179Y^ expressing cells compared to control or mutant p53 knockdown conditions (Fig. 6E).

Importantly, APOE knockdown also affected lipid droplet dynamics in a mutation-specific manner. In p53^H179R^ cells, intracellular lipid droplet numbers increased modestly, although overall neutral lipid content was lower (Supplementary Fig. 7C). In p53^H179Y^ cells, lipid droplet numbers also increased, but total neutral lipid levels remained unchanged, likely reflecting the combined effects of ongoing lipid synthesis and enhanced extracellular fatty acid uptake in these cells (Supplementary Fig. 7D). These results suggest that APOE coordinates lipid homoeostasis downstream of H179 mutants. The increase in lipid droplets following APOE depletion may reflect a compensatory accumulation due to impaired cytoskeletal organisation and lipid handling, rather than increased lipid synthesis, as these experiments were conducted without controlled lipid supplementation.

Together, these findings indicate that p53^H179R^ and p53^H179Y^ promote APOE expression to preserve VIM integrity and cytoskeletal organisation, thereby enhancing cell survival and linking mutant p53-driven lipid metabolic reprogramming to EMT-associated cytoskeletal remodelling. APOE acts as a key mediator of H179 mutant phenotypes, its loss disrupts cytoskeletal organisation, alters lipid droplet dynamics, and reduces cell viability.

### p53^H179R^ and p53^H179Y^ mutants promote an invasive phenotype

**Fig. 7:**
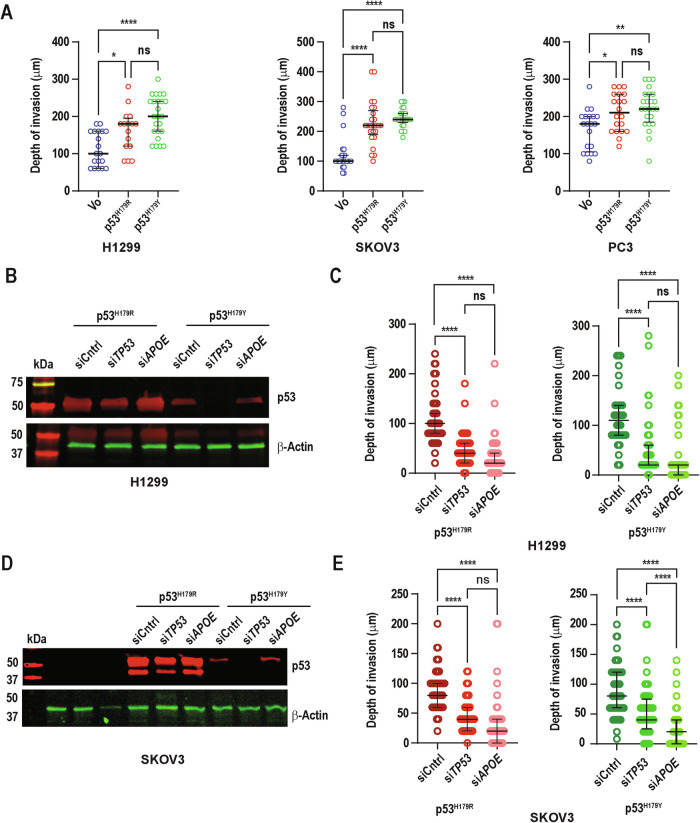
Cancer cells expressing p53^H179R^ and p53^H179Y^ mutants have increased invasive potential. **A** Depth of invasion (μm) of Vo, p53^H179R^ and p53^H179Y^ expressing H1299 (left panel), SKOV3 (middle panel) and PC3 (right panel). **A**, **C**, **E** Z-series of 20 steps at 20μm was collected at 20x. 2% FBS was used as a chemoattract. Data is from Fields: *n* = 4, replicates: *n* = 6, Experiment *n* = 2. Dots represent data from each field. Middle bar represents median and the whiskers represent the 25th and 75th percentile. Significance was determined using One-way ANOVA with Tukey’s multiple comparison test. *p* < 0.05 was considered significant. * - *p* < 0.032, ** - *p* < 0.0021 and ns non-significant. **B**, **D**. Western blot using anti-p53 antibody on protein lysates from H1299 p53^H179R^ and p53^H179Y^ expressing cells treated for 96 h with either control siRNA (siCntrl – 20 nM), siRNA targeting *TP53* (si*TP53*-20nM) or siRNA targeting *APOE* (si*APOE* – 5 nM). Anti-β Actin is blotted using anti-β Actin and used as a loading control. **B** H1299 and **D** SKOV3.

Given our observation that p53^H179R^ and p53^H179Y^ mutants increase intracellular lipid levels as well as the expression of APOE and the mesenchymal marker VIM, we next investigated whether these mutants enhance cancer cell invasiveness. Using an inverted collagen invasion assay, we observed that H1299, SKOV3, and PC3 cells expressing p53^H179R^ or p53^H179Y^ exhibited a significant increase in invasion compared to vector-only (Vo) controls (Fig. 7A and Supplementary Fig. 8A).

To determine whether mutant p53 is required to maintain this invasive phenotype, we transiently knocked down p53^H179R^ and p53^H179Y^ in lung and ovarian cancer cell lines. Silencing either mutant significantly reduced invasion in H1299 and SKOV3 cells (Fig. 7B–E and Supplementary Fig. 9), demonstrating that continued expression of mutant p53 is required to sustain invasiveness. Notably, knockdown of APOE phenocopied the effects of mutant p53 depletion, resulting in a marked decrease in invasiveness.

Together, these data establish that p53^H179R^ and p53^H179Y^ mutants promote cancer cell invasion through an APOE-dependent programme that reinforces VIM organisation and mesenchymal traits. This invasive phenotype represents a functional consequence of mutant p53 driven lipid metabolic reprogramming and cytoskeletal remodelling, linking H179 mutations to aggressive tumour behaviour.

## Discussion

The spectrum of *TP53* mutations in human tumours is remarkable for its diversity and tissue specificity. Despite decades of research there is a paucity of information on the biological implications of mutations beyond the known hotspots. This lack of knowledge makes the use of *TP53* status to effectively improve patient care challenging in a clinical setting. Studies with isogenic cell line and animal models expressing either individual mutant, mutagenesis screens using a cDNA library of mutant p53 [47, 48, 50] or a doxycycline inducible lentiviral CRISPR platform to remove endogenous mutant *TP53* [51] have been conducted. Despite this large amount of work, there is little information on the biological implication of ~75% of missense mutations, of these are the mutations at codon 179 of p53. In this study, we provide the first comprehensive characterisation of *TP53*^*H179R*^ and *TP53*^*H179Y*^ mutations, linking these variants to distinct molecular phenotypes and adverse clinical outcomes.

Analysis of pan-cancer datasets from TCGA and MSK-IMPACT demonstrated that *TP53*^*H179*^ mutations occur across multiple tumour types at frequencies comparable to established hotspot mutations in specific tumour types. These mutations were associated with significantly poorer disease-free survival compared to hotspot, frameshift, nonsense, and *TP53*^*R175H*^ mutations, although these analyses are limited by tumour heterogeneity and potential confounding factors. These data indicate that *TP53*^*H179*^ mutations represent clinically relevant alterations warranting further functional investigation. Functionally, p53^H179R^ and p53^H179Y^ mutants are defective in canonical wild-type p53 tumour suppressor activities. In p53-null cellular backgrounds, these mutants fail to bind canonical p53 response elements, activate classical target genes such as *CDKN1A* and *MDM2*, or suppress cell death, despite retaining the ability to form tetramers, localise to the nucleus, and exhibit high protein stability. Molecular dynamics simulations indicate that substitution of H179, a zinc-coordinating residue within the DNA-binding domain [30], disrupts local zinc coordination and increases structural flexibility, particularly in loops L2 and L3. These structural perturbations provide a plausible explanation for the impaired binding to canonical p53 response elements. Consistent with reports for *TP53*^*R175H*^ and other missense variants [51–54], increased conformational flexibility may also enable interactions with non-canonical DNA elements or protein partners, although such interactions were not directly examined in this study.

Transcriptomic profiling revealed that p53^H179R^ and p53^H179Y^ mutants reprogramme cancer cell biology toward lipid metabolism and epithelial-to-mesenchymal transition (EMT). Both mutants induced expression of genes involved in lipid synthesis, transport, and cytoskeletal remodelling while repressing genes associated with epithelial cell–matrix adhesion. These transcriptional programmes were accompanied by marked accumulation of neutral lipids and increased numbers of lipid droplets across multiple cancer cell types, particularly lung and ovarian cancer models.

Although mutant p53–driven metabolic reprogramming [55] and EMT [56] have been reported previously, these phenotypes are highly mutation, and context-dependent. Several structural p53 mutants, including p53^R175H^ and p53^P151S^, have been shown to promote de novo fatty acid synthesis through inhibition of AMP-activated protein kinase (AMPK) and activation of lipogenic programmes [13]. Similarly, DNA-contact mutants such as p53^R273H^ have been associated with enhanced invasion and metastasis through transcriptional reprogramming [57, 58]. Our analyses reveal that p53^H179R^ primarily drives lipid accumulation through enhanced de novo fatty acid synthesis, associated with increased ACC2 expression, whereas p53^H179Y^ promotes lipid accumulation both by increasing extracellular fatty acid uptake and by enhancing de novo synthesis via reduced inhibitory phosphorylation of ACC1 [37]. Thus, even closely related *TP53* missense mutations engage distinct metabolic routes to converge on lipid accumulation, highlighting the functional heterogeneity of mutant p53–driven metabolic reprogramming.

Importantly, previous studies have largely examined lipid metabolism or EMT as separate outputs of mutant p53 activity. In contrast, our data identify a coordinated programme driven by p53^H179R^ and p53^H179Y^ mutants that links lipid synthesis and, in the case of p53^H179Y^, increased fatty acid uptake to lipid droplet accumulation, APOE upregulation, vimentin (VIM) stabilisation, and enhanced invasion.

A key mechanistic insight from our study is the identification of APOE as a central mediator linking mutant p53 driven lipid metabolic reprogramming to cytoskeletal remodelling and invasion [59–62]. APOE was consistently upregulated at both mRNA and protein levels in p53^H179R/Y^ mutant cells and in tumours harbouring H179 mutations, but not in p53^R175H^. Wild-type p53 has been shown to function not only as a transcriptional activator but also as a transcriptional repressor, through both direct and indirect mechanisms [63]. Direct p53-mediated repression has been reported at promoters of genes involved in cell cycle progression, survival, and metabolism, including *CCNB1* [64], *CDC25C* [65, 66], *BIRC5* [66], and *MYC* [67], often through recruitment of corepressor complexes or histone deacetylases. In addition, p53 can repress transcription indirectly by antagonising transcription factors such as SP1 [68], NF-Y, and SREBP [69], thereby constraining anabolic and metabolic gene expression programmes. In line with this, wild-type p53 occupied the APOE promoter without activating transcription, whereas loss of binding by H179 mutants correlated with increased APOE expression, consistent with relief of transcriptional repression. Although co-repressor recruitment and chromatin modifications were not directly assessed, these data support a model in which H179 mutants relieve wild-type p53–mediated transcriptional restraint, distinct from canonical gain-of-function activation. In this study, we use the term “gain-of-function” to describe mutant p53–dependent phenotypes that exceed those observed in p53-null cells, without implying neomorphic DNA binding or direct transcriptional activation unless explicitly stated. Notably, APOE induction was not observed in p53^R175H^-expressing cells in our models or in previously reported colorectal cancer models, whereas p53^R273H^ has been shown to promote APOE expression and metastasis in vivo [70]. These findings suggest that APOE-dependent coupling of lipid metabolism to cytoskeletal remodelling is not a universal feature of mutant p53 but may define a subset of missense variants with increased conformational flexibility within the DNA-binding domain.

Beyond its established extracellular role in lipid transport, intracellular accumulation of APOE contributed to stabilisation and organisation of VIM filaments, supporting cytoskeletal integrity, cell survival, and invasive behaviour [48]. Disruption of APOE resulted in VIM cleavage, cytoskeletal disorganisation, reduced cell viability, and impaired invasion [49], indicating that APOE is required to maintain mesenchymal cytoskeletal architecture downstream of mutant p53. Importantly, APOE knockdown uncoupled lipid storage from cytoskeletal integrity in a mutation-specific manner: both p53^H179R^ and p53^H179Y^ cells exhibited increased lipid droplets, while overall neutral lipid content was reduced in p53^H179R^ cells and unchanged in p53^H179Y^ cells. Consistently, p53^H179R/Y^ driven invasion, VIM organization, and lipid accumulation all depended on sustained expression of mutant p53 and APOE, as knockdown of either disrupted these phenotypes. Importantly, in our stable cell line models (H1299, SKOV3, and PC3), the expression levels of the H179 mutants are not identical. Despite this, the overall trends in lipid accumulation, APOE upregulation, VIM stabilisation, and increased invasion are consistent, although some quantitative differences may reflect differential protein expression. This represents a limitation of the current models and highlights the need for future studies using more controlled expression systems to precisely compare the functional impact of individual p53 mutants.

Supporting these experimental findings, pan-cancer analyses demonstrated that tumours harbouring *TP53*^*H179*^ mutations exhibit higher APOE expression and poorer disease-free survival compared to p53-null tumours and tumours with *TP53*^*R175H*^ mutations. Elevated APOE levels have been linked to enhanced proliferation, survival, migration, and immune evasion in multiple cancer types [71], providing a plausible clinical context for these associations. Together, these results reinforce that *TP53* mutations are not functionally equivalent to p53 loss. While p53^H179^ mutants retain structural features such as tetramerization and nuclear localisation, they lose canonical tumour suppressor activity and actively drive distinct transcriptional and metabolic programmes, representing a coordinated gain-of-function. This highlights the need for nuanced clinical evaluation of *TP53* status, as treating all mutations as equivalent to p53 inactivation overlooks mutation-specific effects that shape tumour progression and patient outcomes.

**Fig. 8:**
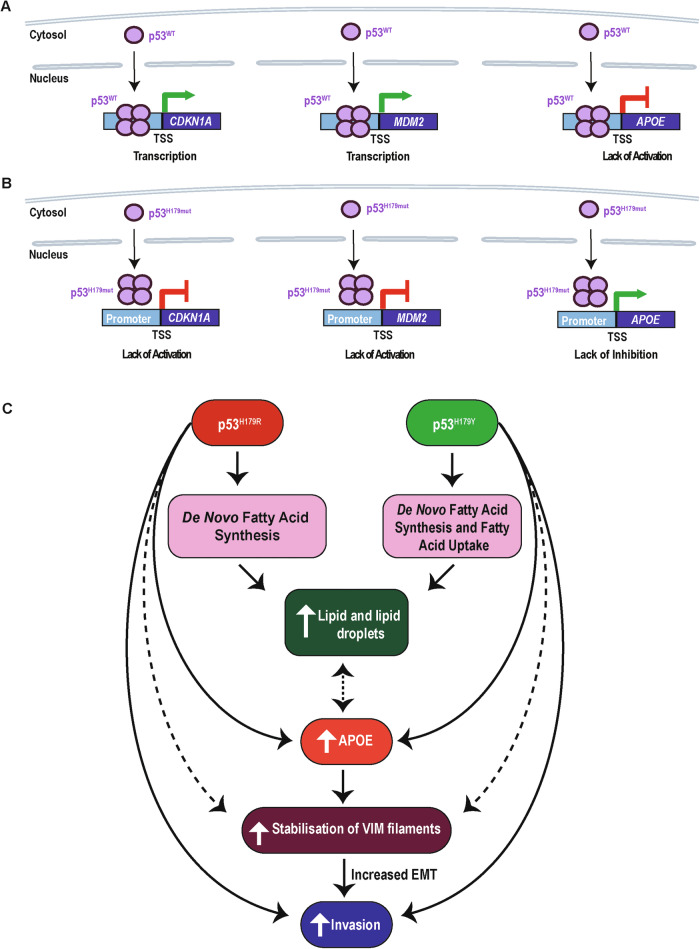
Model illustrating how p53^H179R/Y^ mutants promote lipid reprogramming and invasion. **A** p53^WT^ forms tetramers and binds promoters of canonical p53 targets (e.g., *MDM2* and *CDKN1A*) and *APOE*, leading to induction or repression of transcription of these genes. **B** p53^H179^ mutant forms tetramers but does not bind promoters of canonical p53 target genes (e.g., *MDM2* and *CDKN1A*) and *APOE*, leading to loss-of-function. **C** p53^H179R^ drives de novo lipid synthesis, whereas p53^H179Y^ drives both de novo synthesis and uptake. Together, p53^H179R/Y^ mutants reprogramme lipid metabolism, induce APOE and VIM, and promote invasion. Solid arrows represent findings supported by our experimental evidence; dotted arrows indicate relationships supported by previously published literature.

Our data support a working model (Fig. 8) in which *TP53*^*H179R/Y*^ mutations disrupt canonical tumour suppressor activity and rewire transcriptional programmes to promote a coordinated metabolic and invasive phenotype. Specifically, loss of wild-type p53 binding to canonical response elements, including the APOE promoter, results in loss of transcriptional activation of canonical p53 target genes such as *CDKN1A* and *MDM2* (Fig. 8A, B). While loss of promoter occupancy may permit increased *APOE* expression in this context, this mechanism alone is unlikely to fully explain the specificity of *APOE* induction observed with H179 mutants, as similar loss of p53-dependent activation would also be expected in p53-null cells or in cells expressing other DNA-binding domain mutants such as R175H. Thus, the selective ability of p53^H179R/Y^ to promote APOE expression suggests additional, mutation-specific mechanisms, potentially involving structural flexibility within the DNA-binding domain that enables engagement with non-canonical regulatory elements or context-dependent cofactors, although these interactions remain to be defined. Loss of wild-type p53 binding to canonical response elements, including the APOE promoter, relieves transcriptional repression (Fig. 8A, B). Structural flexibility within the DNA-binding domain may allow engagement with non-canonical regulatory elements or cofactors, although these interactions remain to be defined.

Functionally, p53^H179R^ promotes de novo fatty acid synthesis, whereas p53^H179Y^ additionally enhances uptake of extracellular fatty acids, leading to lipid droplet accumulation. Elevated APOE stabilises VIM filaments, supporting cytoskeletal remodelling, cell survival, and invasion (Fig. 8C). Importantly, APOE mediates the coordination between lipid droplet accumulation and cytoskeletal integrity, its depletion increases lipid droplets while disrupting VIM organization and reducing cell viability. Together, these alterations link mutant p53-driven metabolic rewiring to EMT-associated invasion and may contribute to the poor clinical outcomes observed in tumours harbouring *TP53*^H179^ mutations.

In conclusion, our study identifies *TP53*^*H179R*^ and *TP53*^*H179Y*^ mutations as clinically relevant *TP53* variants associated with adverse outcomes and a distinct gain-of-function programme that integrates lipid metabolic rewiring, APOE upregulation, cytoskeletal stabilisation, and invasion. These findings expand the functional landscape of *TP53* mutations beyond canonical hotspots and underscore the importance of mutation-specific biology in shaping tumour behaviour. As metabolic reprogramming is closely linked to epigenetic regulation [72], future studies will assess whether *TP53*^*H179R/Y*^ driven lipid metabolic changes are accompanied by alterations in histone marks associated with epithelial and mesenchymal differentiation, including H3K4me3, H3K9me3, H3K27ac, and H3K27me3. Further studies will be required to determine whether targeting lipid metabolism or APOE-dependent pathways can be exploited therapeutically in tumours harbouring *TP53*^*H179*^ mutations, either alone or in combination with existing treatment modalities.

## Material and methods

### Patient cohorts

Pan-cancer dataset from The Cancer Genome Atlas and the Memorial Sloan-Kettering-IMPACT dataset (MSK-Impact) representing distinct patient cohorts with no overlapping individuals, were used for this study. The frequency of mutation type (missense, frameshift, nonsense, splice and fusions) was determined from both datasets. Missense mutations at codons 175, 220, 245, 248, 273 and 282 [4] constituted the hotspot mutation group. The frequency of *TP53* mutations at individual codons and for mutations at H179 and R175 across multiple tumour types was determined from these two datasets. The frequency of base change resulting in an amino acid change at codon 179 from H > R/Y/L/D/Q/N/P was ascertained from both datasets. Disease free survival (DFS) analysis was performed using the pan-cancer TCGA dataset. DFS analysis was carried out for tumours with H179 mutations compared to the same tumour types that have either hotspot, frameshift and nonsense, splice and R175. DFS analysis was also performed for tumours with either R158, H193, S241, R249, P278 or R280 mutations compared to matched tumour types containing hotspot mutations. Data for all the above analyses were obtained from cBioPortal [73].

### Generation of Vo, *TP53*^*R175H*^, *TP53*^*H179R*^ and *TP53*^*H179Y*^ expression constructs

Site directed mutagenesis was performed using the Q5^®^ Site-Directed Mutagenesis Kit (New England Biolabs) with plasmid pCMV-Neo-Bam p53 wt (plasmid # 16434, kindly gifted by Bert Vogelstein) as the template under the following conditions, initial denaturation for 30 s at 98 °C, followed by 25 cycles of: Denaturation: 98 °C for 10 s, Annealing: R175H - 66 °C, H179R - 72 °C, H179Y – 69 °C for 30 s, Elongation: 72 °C for 4 min 20 s followed by a final elongation of 2 min at 72 °C. Primers used to introduce the mutations are as follows: R175H_Forward: 5’-GTTGTGAGGC**a**CTGCCCCCAC-3’; R175H_Reverse: 5’-CTCCGTCATGTGCTGTGAC-3’, H179R_Forward: 5’-TGCCCCCACC**g**TGAGCGCTGC-3’; H179R_Reverse: 5’-GCGCCTCACAACCTCCGTCATG- 3’, H179Y_Forward: 5’-CTGCCCCCAC**t**ATGAGCGCTG- 3’; H179Y_Reverse: 5’-GCGCCTCACAACCTCCGTCATG- 3’. The Vector only (Vo) plasmid was obtained by treating 1 μg pCMV-Neo-Bam p53 wt plasmid with *BamHI* restriction enzyme at 37 °C for 1 h, following which the product was run on a 1% agarose gel and the pCMV-Neo-Bam with excised *TP53* was cut from the gel and purified using the QIAGEN QIAquick Gel Extraction Kit, as per manufacturer’s instructions (QIAGEN Inc., UK). Following which T4 DNA ligase was used to ligate and construct the pCMV-Neo-Bam vector only (Vo) plasmid.

The Vo or plasmids bearing the desired mutations were introduced into high-efficiency NEB 5-alpha Competent *E. coli* cells (Q5^®^ Site-Directed Mutagenesis Kit). After 30 min of incubation, the plasmid containing *E.coli* cells were heated at 42 °C for 30 s, then were immediately placed on ice for 5 min and were cultured in SOC for 1 h at 37 °C shaking at 220 rpm to allow recovery of the transformed cells. The recovered cells were cultured overnight on Luria Broth agar plates supplemented with 100 μg/ml of ampicillin. Plasmids were propagated from single colonies and extracted using the QIAGEN Mini-Prep protocol (QIAGEN Inc., UK). All mutations were confirmed by DNA sequencing analysis (Supplementary Fig. 2A).

### Cell culture

One wildtype (wt)-*TP53* and three p53 null cell lines were used for this study. *WT-TP53* cell line: A549 (human lung adenocarcinoma) and p53 null cell lines include: H1299 (human lung adenocarcinoma), SKOV3 (human ovarian cancer) and PC3 (human prostate cancer). A549 and SKOV3 were cultured in Dulbecco’s Modified Eagle Medium (DMEM, Life Technologies), H1299 in Roswell Park Memorial Institute 1640 (RPMI 1640, Life Technologies) and PC3 Ham’s F-12K (Kaighn’s) Medium (Life Technologies) respectively. All cells were maintained in their respective media supplemented with 10% foetal bovine serum (FBS) in a 37 °C humidified incubator at 5% CO_2_ unless stated otherwise. All cell lines were validated for authenticity by CellBank Australia (http://www.cellbankaustralia.com/) using STR profiling. All cell lines are regularly tested negative for mycoplasma contamination.

### Transient plasmid transfection

Cells (100,000) were seeded in 2 ml of media supplemented with 10% FBS in a 6 well plate. After overnight incubation, cells were transfected using Lipofectamine 3000 (LF3000, Invitrogen) with 600 ng of either the Vo or, *TP53*^*WT*^*, TP53*^*R175H*^, *TP53*^*H179R*^ and *TP53*^*H179Y*^. LF3000 (2.5 µl) was diluted in 100 µl of OptiMem (Life Technologies), mixed and incubated for 5 min. In parallel, the plasmid was added to 100 µl of OptiMem along with 2.5 µl of P3000 and combined with the tube containing LF3000 and incubated for a further 10 min. 200 µl of the lipoplexes formed were added to the cells. Media was changed 24 h post transfection following which the cells were fixed at 96 h for immunofluorescence or were harvested for western blot as described below.

### Generation of H1299, SKOV3 and PC3 cell lines stably expressing Vo, p53^*R175H*^, p53^*H179R*^ and p53^*H179Y*^

To generate stable cells, 100,000 cells of H1299, SKOV3 and PC3 cells were plated in 6-well plates overnight following which they were transfected using Lipofectamine 3000 (Life technologies, L3000015) and 600 ng of the Vo or *TP53*^*R175H*^, *TP53*^*H179R*^ and *TP53*^*H179Y*^ encoding plasmids using the protocol described for transient plasmid transfection. 24 h post-transfection, media containing 10% FBS and 1 mg/ml of G-418 (Life technologies) for H1299 and PC3 and 4 mg/ml of G-418 (Life technologies) for SKOV3 was replaced the following day. In parallel, there was a well with no plasmid transfection which was also treated similarly. Cell growth was followed and media containing selection antibiotic was changed every 2–3 days for up to two weeks or until colonies of cell appear in the transfected wells and there are no surviving cells in the untransfected cells remaining. Surviving cells were then established as independent cell lines and expression of Vo, p53^*R175H*^, p53^*H179R*^ and p53^*H179Y*^ was confirmed routinely using western blot. The stable cells were maintained in their respective media supplemented with 10% foetal bovine serum (FBS) and 1 mg/ml of G-418 in a 37 °C humidified incubator at 5% CO_2_ unless stated otherwise.

### siRNA transfection

Cells were reverse transfected with stealth modified 25 bp duplex siRNA targeted to p53 (si-p53 5′- GACGCUAGGAUCUGACUGCGGCUCC-3′), *APOE* (siAPOE-1 5’-UUCAACUCCUUCAUGGUCUCG-3’, siAPOE-2: 5’-UUUGUAGGCCUUCAACUCCUU-3’) [74] and a control siRNA (siCntrl 5’ - CCACACGAGUCUUACCAAGUUGCUU-3′) with no known human mRNA targets. Stealth siRNAs were transfected at a final concentration of 20 nM (siCntrl and si*TP53*) or 5 nM (2.5 nM: si*APOE* and 2.5 nM si*APOE2*) using Lipofectamine RNAiMax (Life Technologies). Both siRNAs and RNAiMax were diluted in OptiMem. After 5 min at room temperature, the diluted RNAiMax was added to the siRNAs, and the mixture was incubated for a further 10 min. The lipoplexes formed were added to cells. After overnight transfection, the culture medium was replaced with media supplemented with 10% FBS. Cells were fixed at 96 h for immunofluorescence or were harvested for western blot as described below.

### Western blot and glutaraldehyde crosslinking and media

Protein lysates were prepared from H1299, SKOV3 and PC3 cells either stably or transiently expressing Vo, p53^*R175H*^, p53^*H179R*^ and p53^*H179Y*^, or treated with siCntrl, si*TP53* or si*APOE*. Cells were lysed in RIPA lysis buffer (Thermo Fisher Scientific, 89901). Total protein concentration was determined using the Pierce BCA kit (Thermo Fisher Scientific) or using the Qubit™ Protein and Protein Broad Range (BR) Assay Kit (Thermo Fisher Scientific). After total protein lysates were obtained, a subset of these were used for oligomerisation assays. Crosslinking was performed as previously established [75–77] by adding, 0.1% (v/v) glutaraldehyde to 50 μg of protein lysate and incubating at room temperature for 15 min prior to denaturation. To 50 to 100 μg of protein lysate, 100 mM DTT and 4x Laemmli buffer was added, and this mix was denatured either at 5 min at 95 °C or 10 min at 70 °C depending on the protein of interest. Proteins were separated by electrophoresis on 4–15% Mini-PROTEAN® precast protein gels (Bio-Rad). Proteins were transferred onto nitrocellulose membranes using the Mini Trans-Blot® Electrophoretic Transfer Cell and transfer stacks (Bio-Rad). Efficiency of electrophoretic transfer of SDS-denatured proteins to nitrocellulose membranes and protein loading in case of secreted APOE media, were assessed by staining the membrane with Ponceau S solution (ab270042) for 5 min at room temperature, followed by rinsing the membrane in distilled water. Membranes were blocked with Odyssey® Blocking Buffer (PBS/TBS) (Licor) for 30 min and then incubated with the primary antibodies (mouse anti-p53 (DO-1) at 1:1000 for 1 h at RT, rabbit anti-APOE (EPR19392) at 1: 1000, overnight at 4 °C, rabbit anti-Acetyl-CoA Carboxylase (3662) at 1:1000, overnight at 4 °C, rabbit anti-Phospho-Acetyl-CoA Carboxylase (3661) at 1:1000, overnight at 4 °C and mouse anti-β-actin at 1:5000 for 1 hr at RT) diluted in Odyssey® Blocking Buffer (PBS/TBS)/0.2% Tween 20 and then incubated for 1 h with the secondary antibodies (IRDye® 680RD Goat anti-Mouse IgG (Licor) and IRDye® 800CW Goat anti-Rabbit IgG (Licor)) diluted in Odyssey® Blocking Buffer (PBS/TBS)/0.2% Tween 20. Membranes were imaged on the Odyssey® CLx Imaging System (Licor) according to the manufacturer’s instructions. Images were quantified using Image Studio (Licor) software.

Secreted APOE was determined from culture media without FBS of H1299 cells Vo, p53^*H179R*^ and p53^*H179Y*^ seeded for 96 h either with or without siRNA treatment. 5 ml of collected culture media was concentrated using Amicon ultra 0.5 ml 10 kDa MWCO filters (Millipore) and protein levels were measured using 10 μl of concentrated media as described above. 80 μg of concentrated protein from media from H1299 stable cell media was separated and blotted as described above. For detection of secreted APOE in culture media from Vo, p53^*H179R*^ and p53^*H179Y*^ stably expressed in PC3 and SKOV3 cells, 5 ml of FBS-free media was collected and the concentration of protein measured as outlined for H1299. 15 μg of concentrated protein from media from SKOV3 and PC3 stable cells were separated and dry blotted using iBlot 2 and iBlot™ Transfer Stack (Thermo Fisher Scientific) at 23 V for 8 min. Western blotting for secreted APOE protein was performed as outlined above.

### Cut & Run

CUT&RUN assays were performed using the CUT&RUN kit from Cell Signalling Technology (#86652) according to the manufacturer’s instructions. H1299 cells were transiently transfected with Vo or, *TP53*^*WT*^*, TP53*^*R175H*^, *TP53*^*H179R*^ and *TP53*^*H179Y*^ and co-transfected with EBFP2 and flow sorted for cells expressing EBFP2 as a marker of transfected cells [78]. The resulting single-cell suspensions were collected (10^5^ cells/reaction), washed, and rotated with Concavalin A magnetic beads for 2 h at 4 °C with 500 μg of anti-p53 antibody (DO-1). Subsequently, the reactions were rotated with pAG-MNase enzyme, which was activated by addition of CaCl_2_ and incubated sequentially for 30 min at 4 °C and 37 °C. The resulting DNA fragments were purified with DNA purification buffers and spin columns (New England Biolabs) according to manufacturer’s instructions. Input samples underwent DNA extraction before purifying DNA and performing qPCR as described below.

### CUT&RUN sequencing, peak-calling, and p53 target gene analysis

For CUT&RUN sequencing, H1299 cells stably expressing Vo, p53^*R175H*^, p53^*H179R*^ and p53^*H179Y*^ along with A549 (p53^WT^) cells were treated with either solvent control (DMSO) or 10 μM of Nutlin-3A for 24 h before performing CUT&RUN as described above. Dual-indexed CUT&RUN libraries were prepared by using a DNA Library Prep Kit for Illumina Systems (Cell Signalling Technology) according to the manufacturer’s instructions. Fastq files generated from one lane of an FCL flow cell on an MGI DNBSEQ-G400 sequencer (Lincoln Genomics; PE150, 502 M unique reads) were demultiplexed by using mgikit v2.0.1 [79] then subjected to QC, mapping (GRCh38), and peak-calling with macs2 v2.2.7.1 by using nf-core/cutandrun v3.2.2 [80, 81] and Nextflow v25.04.6 [82] in narrow peak mode with default settings (FDR q-value threshold 0.01) and CPM normalisation mode (bin size = 1). narrowPeak files of significant peaks for Nutlin-3A and DMSO-treated samples relative to p53-null vector-only controls were intersected by using BEDTools v2.31.0 [83] to retain significant peaks unique to Nutlin-3A treatment, and annotated by using HOMER v5.1annotatePeaks.pl [84]. Signal profile plots were generated from nf-core/cutandrun bigWig output files by using deepTools v3.5.6 computeMatrix and plotProfiles [85]. For p53 target gene analysis, HOMER annotation data for significant peaks was filtered for matches with a list of 473 previously reported p53 target genes [86–89] (Supplementary Table S1). Overlapping peak regions associated with p53 targets in our data were identified by using BEDTools intersect v2.31.0, then used as region inputs to generate signal plots by using deepTools v3.5.6.

### Molecular dynamic simulations

Crystal structure of p53 DBD^WT^ (residues from 94 to 291, PDB ID: 2AHI) was used generate structural models of p53 DBD^H179R^ and DBD^H179Y^. Mutations were introduced in the p53DBD^WT^ structure (PDB ID: 2AHI) in Pymol [90] and both the p53 DBD^WT^ and p53 DBD^H179R/Y^ were subject to MD simulations. The Xleap module of AMBER 18 [91] was used to prepare the systems for the MD simulations. Each simulation system was neutralised with appropriate numbers of counter ions and each neutralised system was solvated in an octahedral box with TIP3P [92] water molecules, leaving at least 10 Å between the solute atoms and the boundaries of the box. MD simulations were carried out with the pmemd.cuda module of the AMBER 18 package in combination with the ff14SB force field [93]. MD simulations were carried out in explicit solvent at 300 K. During the simulations the long-range electrostatic interactions were treated with the particle mesh Ewald [94] method using a real space cut off distance of 9 Å. The settle [95] algorithm was used to constrain bond vibrations involving hydrogen atoms, which allowed a time step of 2 fs during the simulations. Solvent molecules and counter ions were initially relaxed using energy minimisation with restraints on the protein atoms. This was followed by unrestrained energy minimisation to remove any steric clashes. Subsequently the system was gradually heated from 0 to 300 K using MD simulations with positional restraints (force constant: 50 kcal mol^−1^ Å^−2^) on the non-hydrogen protein atoms over a period of 0.25 ns allowing water molecules and ions to move freely followed by gradual removal of the positional restraints and a 2 ns unrestrained equilibration of the whole system at 300 K. The resulting system was used as the starting structure for the production phase and three independent (using different initial random velocities) MD simulations were carried out for 250 ns each. Accelerated MD simulations (aMD) with dual boost potential [96] were carried out for 5μs to further enhance the conformational sampling of the p53 DBDs. Simulation trajectories were visualised using VMD [97] and figures were generated using Pymol [90].

### RNA extraction, cDNA synthesis and RT-qPCR

RNA was extracted from H1299, SKOV3 and PC3 cells stably or transiently expressing Vo, p53^*R175H*^, p53^*H179R*^ and p53^*H179Y*^, or treated with siCntrl, si*TP53* or si*APOE* using PureLink™ RNA Mini Kit (Thermo Fisher Scientific) using the manufacturer’s protocol. Extracted RNA (500 ng, 1 μg or 2 μg) was DNase I treated using DNase I, Amplification Grade (Life Technologies) and reverse-transcribed using qScript cDNA SuperMix (Quanta Biosciences), according to the manufacturer’s instructions. RTqPCR was performed using QuantStudio 12 K Flex Real-Time PCR System (Life Technologies) and SYBR Premix Ex Taq II (Tli RNase H Plus) (Takara Bio). RT-qPCR was performed for each sample and primer pair in duplicate. Primer sequences used are listed in Supplementary Table S2 [98, 99].

### RNA-sequencing

RNA was extracted from H1299 cells stably expressing Vo, p53^*R175H*^, p53^*H179R*^ and p53^*H179Y*^ as described above. 250 ng of total RNA was used to make libraries using the TruSeq Stranded mRNA kit (Illumina) as per the manufacturer’s protocols. Sequencing was done on the Illumina NovaSeq - S4 - PE 150 Cycle (2x150bp, paired end) with 1 pool of 12 libraries performed in 1 lane. Reads were aligned using STAR [100] and GRCh38 version was used as reference index. Alignment scores were between 72 and 81% for all samples. Gene abundance was quantified using HTseqCounts [101]. Differential gene expression was measured using DESeq2 [102]. Pathway analysis was performed using EnrichR [103]. Overlapping genes was determined using Venny 2.1 [104]. Heatmap was generated using Heatmapper [105]. RNA-sequencing data is deposited into the sequence read archive (SRA) database under the accession number PRJNA1422816.

### Immunofluorescence

Cells were fixed in 37 °C 4% paraformaldehyde in 0.1 M PBS. Fixed cells were thoroughly washed in PBS/TBS, permeabilized with 0.2% Triton X-100 (SigmaAldrich) and following further washing with PBS/TBS, were blocked with 1% BSA in PBS/TBS with 0.2% cold fish skin gelatin (SigmaAldrich) prior to incubation with primary antibodies in blocking solution for 1 h at room temperature or overnight at 4 °C. Following primary incubation, cells were again washed thoroughly with 0.1%TWEEN-20 (SigmaAldrich) in PBS/TBS to remove any unbound primary antibody and then incubated with a fluorophore-labelled secondary antibody at 4 °C for 90 min. Sequential immunolabelling was carried out in some instances, whereby cells were thoroughly washed with 0.1%TWEEN-20 (SigmaAldrich) in PBS/TBS and re-blocked prior to incubation with a second primary antibody. Neutral lipids were labelled with BODIPY™ 493/503 (1:1000 in PBS, Thermo Fisher Scientific) for 15 min at room temperature. The DNA-binding dye Hoescht 33258 (Molecular Probes, 2 μg/mL) was used to visualise cell nuclei. Cells were thoroughly washed in PBS/TBS and a final wash with distilled water prior to mounting in Fluoromount-G (SouthernBiotech) on glass slides.

### De novo lipid synthesis and fatty acid uptake assay

For de novo fatty acid synthesis, the cells were seeded at a density of 10,000 cells per well in a 24 well plate on coverslips. The cells were serum starved the following day for 60 min and 90 min following which they were fixed with 4% paraformaldehyde. The cells were then stained with BODIPY™ 493/503 (1:1000 in PBS, Thermo Fisher Scientific) for 15 min at room temperature. De novo synthesis was assessed in the serum starved cells for 60 min and 90 min which were transiently transfected with either control siRNA or *TP53* targeted siRNA for 72 h.

Fatty acid uptake assay was performed using the Fatty Acid Uptake Assay Kit from Abcam (ab287857). Cells were seeded at a density of 50,000 cells per well in a black clear bottom 96 well plate and left overnight. The cells were then serum starved for 60 min the following day. Fatty acid stock assay was diluted in buffer (1:500) and 100 μL fluorescent fatty acid analogue was added per well. Kinetics of fatty acid uptake was then measured for 30 min with fluorescence recorded at 1 min interval at Ex/Em = 488/523 nm using Victor X4 (Perkin Elmer) spectrophotometer using the bottom read function. Fatty acid uptake was assessed in the same way for serum starved cells for 60 min which were transiently transfected with either control siRNA or *TP53* targeted siRNA for 72 h.

### Antibodies

The following primary antibodies were used: mouse anti-p53 monoclonal to N-terminal of p53 (DO-1, MA5-12571, Thermo Fisher Scientific, 1:1000/1:300); rabbit anti-Histone H3 monoclonal (EPR17785, ab201456, Abcam, 1:5000); rabbit anti-β Actin monoclonal (SP124, ab115777, Abcam, 1:5000); rabbit anti-Lamin B1 polyclonal (ab16048, Abcam, 1:1000); rabbit anti-Vimentin monoclonal (EPR3776, ab92547, abcam, 1:300/1:1000); rabbit anti-APOE monoclonal (EPR19392, ab183597, abcam, 1:1000/1:250); rabbit anti-Acetyl-CoA Carboxylase polyclonal (3662, Cell Signalling Technology 1:1000); rabbit anti-Phospho-Acetyl-CoA Carboxylase polyclonal (3661, Cell Signalling Technology 1:1000); rabbit anti-Vinculin monoclonal (VPA00888, Biorad, 1:5000). Secondaries used were goat anti-mouse Alexa Fluor 488 (Molecular Probes, 1:1000) goat anti-mouse Alexa Fluor 568 (Molecular Probes, 1:1000) and goat anti-rabbit Alexa Flour 568 (Molecular Probes, 1:000).

### Inverted collagen assay

Inverted collagen assay [50] was performed in 24 well plates. Rat tail collagen I (Thermo Fisher Scientific) was diluted to 2 mg/ml as per manufacturer’s instructions. 50,000 cells/well were plated overnight. Following which the culture media was removed and 350 μl of 2 mg/ml of Rat tail collagen I (Thermo Fisher Scientific) prepared as per manufacturer instructions was added to each well. The gel was allowed to polymerise at 37 °C in a CO_2_ incubator with humidifier for 3 h, and then 1 mL of media supplemented with 2% FBS as a chemoattractant was added to the plate to cover the gel. The ability of the cells to invade vertically was investigated at 72 h post addition of gel. At the end of each experiment, the cells were fixed with 4% paraformaldehyde for 15 min at 37 °C and subsequently stained with Hoescht 33258 (Molecular Probes) solution (50 ng/ml) at room temperature for twenty minutes. The fixed cells were imaged using a the BioTek Lionheart Imager (Agilent). Each well was imaged at 4 unique locations, taking 200–400 μm z-stack images for each location. Image was processed using the BioTek Gen 5 software (Agilent).

### Imaging and quantification

Imaging and quantification of lipid, APOE and Vimentin was performed using Lionheart FX automated Imager (Biotek) and GEN5 sofrware, (version 3.1). Representative images were obtained using Nikon A1 confocal microscope with 60X oil immersion objective. Quantification of lipid and lipid droplets was performed using a published analysis pipeline with CellProfiler [106].

### Statistics

Significance for disease free survival was determined using log rank test and *p* < 0.05 was significant. Log rank t-test, Mann-Whitney, Kruskal-Wallis, ordinary one way ANOVA, Welch’s and unpaired t-tests were used along with Tukey’s multiple comparison test. Graph Pad Prism was used to perform these analyses.

### Ethics approval and consent to participate

We confirm that all methods were performed in accordance with the relevant guidelines and regulations. Ethics approval was not required for this study as all experimental work was conducted exclusively using commercially available human cancer cell lines. These cell lines were genetically modified in house using plasmid-based approaches to introduce *TP53* mutant constructs and did not involve the derivation of new human material. All patient data were accessed via cBioPortal and comprised publicly available, de‑identified datasets.

## Supplementary information

Supplementary Figure 1

Supplementary Figure 2

Supplementary Figure 3

Supplementary Figure 4

Supplementary Figure 5

Supplementary Figure 6

Supplementary Figure 7

Supplementary Figure 8

Supplementary Figure 9

Supplementary Figure Legends

Supplementary Table S1

Supplementary Table S2

## Data Availability

Data generated in the present study will be made available on request from the corresponding author.

## References

[1] Lane DP. Cancer. p53, guardian of the genome. Nature. 1992;358:15–6. 1614522 10.1038/358015a0

[2] Lang GA, Iwakuma T, Suh Y-A, Liu G, Rao VA, Parant JM, et al. Gain of function of a p53 hot spot mutation in a mouse model of Li-Fraumeni syndrome. Cell. 2004;119:861–72. 15607981 10.1016/j.cell.2004.11.006

[3] Olive KP, Tuveson DA, Ruhe ZC, Yin B, Willis NA, Bronson RT, et al. Mutant p53 gain of function in two mouse models of Li-Fraumeni syndrome. Cell. 2004;119:847–60. 15607980 10.1016/j.cell.2004.11.004

[4] Hainaut P, Hollstein M. p53 and human cancer: the first ten thousand mutations. In: Advances in cancer research. Elsevier, 1999, p. 81–137. 10.1016/s0065-230x(08)60785-x 10549356

[5] Leroy B, Anderson M, Soussi T. TP53 mutations in human cancer: database reassessment and prospects for the next decade. Hum Mutat. 2014;35:672–88. 24665023 10.1002/humu.22552

[6] Donehower LA, Soussi T, Korkut A, Liu Y, Schultz A, Cardenas M, et al. Integrated analysis of TP53 gene and pathway alterations in the cancer genome Atlas. Cell Rep. 2019;28:1370–84.e5. 31365877 10.1016/j.celrep.2019.07.001 PMC7546539

[7] Kastenhuber ER, Lowe SW. Putting p53 in context. Cell. 2017;170:1062–78. 28886379 10.1016/j.cell.2017.08.028 PMC5743327

[8] Xu J, Wang J, Hu Y, Qian J, Xu B, Chen H, et al. Unequal prognostic potentials of p53 gain-of-function mutations in human cancers associate with drug-metabolizing activity. Cell Death Dis. 2014;5:e1108. 24603336 10.1038/cddis.2014.75 PMC3973211

[9] Parikh N, Hilsenbeck S, Creighton CJ, Dayaram T, Shuck R, Shinbrot E, et al. Effects of TP53 mutational status on gene expression patterns across 10 human cancer types. J Pathol. 2014;232:522–33. 24374933 10.1002/path.4321 PMC4362779

[10] Wang X, Sun Q. TP53 mutations, expression and interaction networks in human cancers. Oncotarget. 2016;8:624–43. 10.18632/oncotarget.13483 PMC5352183 27880943

[11] Malekzadeh P, Pasetto A, Robbins PF, Parkhurst MR, Paria BC, Jia L, et al. Neoantigen screening identifies broad TP53 mutant immunogenicity in patients with epithelial cancers. J Clin Investig. 2019;129:1109–14. 30714987 10.1172/JCI123791 PMC6391139

[12] Freed-Pastor WA, Mizuno H, Zhao X, Langerød A, Moon SH, Rodriguez-Barrueco R, et al. Mutant p53 disrupts mammary tissue architecture via the mevalonate pathway. Cell. 2012;148:244–58. 22265415 10.1016/j.cell.2011.12.017 PMC3511889

[13] Zhou G, Wang J, Zhao M, Xie TX, Tanaka N, Sano D, et al. Gain-of-function mutant p53 promotes cell growth and cancer cell metabolism via inhibition of AMPK activation. Mol Cell. 2014;54:960–74. 24857548 10.1016/j.molcel.2014.04.024 PMC4067806

[14] Ingallina E, Sorrentino G, Bertolio R, Lisek K, Zannini A, Azzolin L, et al. Mechanical cues control mutant p53 stability through a mevalonate-RhoA axis. Nat Cell Biol. 2018;20:28–35. 29255172 10.1038/s41556-017-0009-8 PMC6179142

[15] Tombari C, Zannini A, Bertolio R, Pedretti S, Audano M, Triboli L, et al. Mutant p53 sustains serine-glycine synthesis and essential amino acids intake promoting breast cancer growth. Nat Commun. 2023;14:6777. 37880212 10.1038/s41467-023-42458-1 PMC10600207

[16] Tombari C, Zannini A, Bertolio R, Pedretti S, Audano M, Triboli L, et al. Mutant p53 sustains serine-glycine synthesis and essential amino acids intake promoting breast cancer growth. Nat Commun. 2023;14:7129. 37880212 10.1038/s41467-023-42458-1 PMC10600207

[17] Butler JS, Loh SN. Structure, function, and aggregation of the zinc-free form of the p53 DNA binding domain. Biochemistry. 2003;42:2396–403. 12600206 10.1021/bi026635n

[18] Hingorani SR, Wang L, Multani AS, Combs C, Deramaudt TB, Hruban RH, et al. Trp53R172H and KrasG12D cooperate to promote chromosomal instability and widely metastatic pancreatic ductal adenocarcinoma in mice. Cancer Cell. 2005;7:469–83. 15894267 10.1016/j.ccr.2005.04.023

[19] Liu G, McDonnell TJ, Montes de Oca Luna R, Kapoor M, Mims B, El-Naggar AK, et al. High metastatic potential in mice inheriting a targeted p53 missense mutation. Proc Natl Acad Sci USA. 2000;97:4174–9. 10760284 10.1073/pnas.97.8.4174 PMC18187

[20] Correction for Morton. Mutant p53 drives metastasis and overcomes growth arrest/senescence in pancreatic cancer. Proc Natl Acad Sci USA. 2022;119:e2204610119. 35471899 10.1073/pnas.2204610119 PMC9170066

[21] Capaci V, Bascetta L, Fantuz M, Beznoussenko GV, Sommaggio R, Cancila V, et al. Mutant p53 induces Golgi tubulo-vesiculation driving a prometastatic secretome. Nat Commun. 2020;11:3945. 32770028 10.1038/s41467-020-17596-5 PMC7414119

[22] Kogan-Sakin I, Tabach Y, Buganim Y, Molchadsky A, Solomon H, Madar S, et al. Mutant p53(R175H) upregulates Twist1 expression and promotes epithelial-mesenchymal transition in immortalized prostate cells. Cell Death Differ. 2011;18:271–81. 20689556 10.1038/cdd.2010.94 PMC3131887

[23] Yeudall WA, Vaughan CA, Miyazaki H, Ramamoorthy M, Choi MY, Chapman CG, et al. Gain-of-function mutant p53 upregulates CXC chemokines and enhances cell migration. Carcinogenesis. 2012;33:442–51. 22114072 10.1093/carcin/bgr270

[24] Escoll M, Gargini R, Cuadrado A, Anton IM, Wandosell F. Mutant p53 oncogenic functions in cancer stem cells are regulated by WIP through YAP/TAZ. Oncogene. 2017;36:3515–27. 28166194 10.1038/onc.2016.518

[25] Solomon H, Dinowitz N, Pateras IS, Cooks T, Shetzer Y, Molchadsky A, et al. Mutant p53 gain of function underlies high expression levels of colorectal cancer stem cells markers. Oncogene. 2018;37:1669–84. 29343849 10.1038/s41388-017-0060-8 PMC6448595

[26] Chen S, Wang Q, Yu H, Capitano ML, Vemula S, Nabinger SC, et al. Mutant p53 drives clonal hematopoiesis through modulating epigenetic pathway. Nat Commun. 2019;10:5649. 31827082 10.1038/s41467-019-13542-2 PMC6906427

[27] Ali A, Wang Z, Fu J, Ji L, Liu J, Li L, et al. Differential regulation of the REGγ-proteasome pathway by p53/TGF-β signalling and mutant p53 in cancer cells. Nat Commun. 2013;4:2667. 24157709 10.1038/ncomms3667 PMC3876931

[28] Kolukula VK, Sahu G, Wellstein A, Rodriguez OC, Preet A, Iacobazzi V, et al. SLC25A1, or CIC, is a novel transcriptional target of mutant p53 and a negative tumor prognostic marker. Oncotarget. 2014;5:1212–25. 24681808 10.18632/oncotarget.1831 PMC4012738

[29] Alam SK, Yadav VK, Bajaj S, Datta A, Dutta SK, Bhattacharyya M, et al. DNA damage-induced ephrin-B2 reverse signaling promotes chemoresistance and drives EMT in colorectal carcinoma harboring mutant p53. Cell Death Differ. 2016;23:707–22. 26494468 10.1038/cdd.2015.133 PMC4986638

[30] Blanden AR, Yu X, Blayney AJ, Demas C, Ha JH, Liu Y et al. Zinc shapes the folding landscape of p53 and establishes a pathway for reactivating structurally diverse cancer mutants. Elife. 2020;9:e61487. 10.7554/eLife.61487 PMC7728444 33263541

[31] Weinstein JN, Collisson EA, Mills GB, Shaw KRM, Ozenberger BA, Ellrott K, et al. The cancer genome atlas pan-cancer analysis project. Nat Genet. 2013;45:1113. 24071849 10.1038/ng.2764 PMC3919969

[32] Zehir A, Benayed R, Shah RH, Syed A, Middha S, Kim HR, et al. Mutational landscape of metastatic cancer revealed from prospective clinical sequencing of 10,000 patients. Nat Med. 2017;23:703–13. 28481359 10.1038/nm.4333 PMC5461196

[33] Malecka KA, Ho WC, Marmorstein R. Crystal structure of a p53 core tetramer bound to DNA. Oncogene. 2009;28:325–33. 18978813 10.1038/onc.2008.400 PMC2629805

[34] Vogel FCE, Chaves-Filho AB, Schulze A. Lipids as mediators of cancer progression and metastasis. Nat Cancer. 2024;5:16–29. 38273023 10.1038/s43018-023-00702-z

[35] Olzmann JA, Carvalho P. Dynamics and functions of lipid droplets. Nat Rev Mol Cell Biol. 2019;20:137–55. 30523332 10.1038/s41580-018-0085-z PMC6746329

[36] Petan T, Jarc E, Jusović M. Lipid droplets in cancer: guardians of fat in a stressful World. *Molecules* 2018;23:1941. 10.3390/molecules23081941 PMC6222695 30081476

[37] Wang Y, Yu W, Li S, Guo D, He J, Wang Y. Acetyl-CoA carboxylases and diseases. Front Oncol. 2022;12:836058. 35359351 10.3389/fonc.2022.836058 PMC8963101

[38] Huang Y, Mahley RW. Apolipoprotein E: structure and function in lipid metabolism, neurobiology, and Alzheimer’s diseases. Neurobiol Dis. 2014;72:3–12. 25173806 10.1016/j.nbd.2014.08.025 PMC4253862

[39] Lerma Clavero A, Boqvist PL, Ingelshed K, Bosdotter C, Sedimbi S, Jiang L, et al. MDM2 inhibitors, nutlin-3a and navtemadelin, retain efficacy in human and mouse cancer cells cultured in hypoxia. Sci Rep. 2023;13:4583. 36941277 10.1038/s41598-023-31484-0 PMC10027891

[40] Elliott DA, Halliday GM, Garner B. Apolipoprotein-E forms dimers in human frontal cortex and hippocampus. BMC Neurosci. 2010;11:23. 20170526 10.1186/1471-2202-11-23 PMC2837047

[41] Strickland MR, Rau MJ, Summers B, Basore K, Wulf J 2nd, Jiang H, et al. Apolipoprotein E secreted by astrocytes forms antiparallel dimers in discoidal lipoproteins. Neuron. 2024;112:1100–1109.e5. 38266643 10.1016/j.neuron.2023.12.018 PMC10994765

[42] Wang H, Du S, Cai J, Wang J, Shen X. Apolipoprotein E2 promotes the migration and invasion of pancreatic cancer cells via activation of the ERK1/2 Signaling Pathway. Cancer Manag Res. 2020;12:13161–71. 33376407 10.2147/CMAR.S284115 PMC7764636

[43] Helfand BT, Mendez MG, Murthy SN, Shumaker DK, Grin B, Mahammad S, et al. Vimentin organization modulates the formation of lamellipodia. Mol Biol Cell. 2011;22:1274–89. 21346197 10.1091/mbc.E10-08-0699 PMC3078081

[44] Berr AL, Wiese K, Dos Santos G, Koch CM, Anekalla KR, Kidd M, et al. Vimentin is required for tumor progression and metastasis in a mouse model of non-small cell lung cancer. Oncogene. 2023;42:2074–87. 37161053 10.1038/s41388-023-02703-9 PMC10275760

[45] Tadokoro A, Kanaji N, Liu D, Yokomise H, Haba R, Ishii T, et al. Vimentin regulates invasiveness and is a poor prognostic marker in non-small cell lung cancer. Anticancer Res. 2016;36:1545–51. 27069130

[46] Gonzalez VD, Samusik N, Chen TJ, Savig ES, Aghaeepour N, Quigley DA, et al. Commonly occurring cell subsets in high-grade serous ovarian tumors identified by single-cell mass cytometry. Cell Rep. 2018;22:1875–88. 29444438 10.1016/j.celrep.2018.01.053 PMC8556706

[47] Burch TC, Watson MT, Nyalwidhe JO. Variable metastatic potentials correlate with differential plectin and vimentin expression in syngeneic androgen independent prostate cancer cells. PLoS ONE. 2013;8:e65005. 23717685 10.1371/journal.pone.0065005 PMC3661497

[48] Battaglia RA, Delic S, Herrmann H, Snider NT. Vimentin on the move: new developments in cell migration. F1000Res. 2018;7:F1000-Faculty. 10.12688/f1000research.15967.1 PMC6241562 30505430

[49] Zhang L, Wang J, Yan Y, Xiang L, Zhai X, Cai L, et al. Vimentin fragmentation and its role in amyloid-beta plaque deposition in Alzheimer’s Disease. Int J Mol Sci. 2025;26:2857. 40243407 10.3390/ijms26072857 PMC11988971

[50] McArdle TJ, Ogle BM, Noubissi FK. An in vitro inverted vertical invasion assay to avoid manipulation of rare or sensitive cell types. J Cancer. 2016;7:2333–40. 27994672 10.7150/jca.15812 PMC5166545

[51] Wang Z, Burigotto M, Ghetti S, Vaillant F, Tan T, Capaldo BD, et al. Loss-of-function but not gain-of-function properties of mutant TP53 are critical for the proliferation, survival, and metastasis of a broad range of cancer cells. Cancer Discov. 2024;14:362–79. 37877779 10.1158/2159-8290.CD-23-0402 PMC10850947

[52] Giacomelli AO, Yang X, Lintner RE, McFarland JM, Duby M, Kim J, et al. Mutational processes shape the landscape of TP53 mutations in human cancer. Nat Genet. 2018;50:1381–7. 30224644 10.1038/s41588-018-0204-y PMC6168352

[53] Kotler E, Shani O, Goldfeld G, Lotan-Pompan M, Tarcic O, Gershoni A, et al. A Systematic p53 mutation library links differential functional impact to cancer mutation pattern and evolutionary conservation. Mol Cell. 2018;71:178–190.e8. 29979965 10.1016/j.molcel.2018.06.012

[54] Boettcher S, Miller PG, Sharma R, McConkey M, Leventhal M, Krivtsov AV, et al. A dominant-negative effect drives selection of TP53 missense mutations in myeloid malignancies. Science. 2019;365:599–604. 31395785 10.1126/science.aax3649 PMC7327437

[55] Koo KY, Moon K, Song HS, Lee M-S. Metabolic regulation by p53: Implications for cancer therapy. Mol Cells. 2025;48:100198. 39986611 10.1016/j.mocell.2025.100198 PMC11925517

[56] Semenov O, Daks A, Fedorova O, Shuvalov O, Barlev NA. Opposing roles of wild-type and mutant p53 in the process of epithelial to mesenchymal transition. Front Mol Biosci. 2022;9:928399. 10.3389/fmolb.2022.928399 PMC9261265 35813818

[57] Meng W, Yu S, Li Y, Wang H, Feng Y, Sun W, et al. Mutant p53 achieves function by regulating EGR1 to induce epithelial mesenchymal transition. Tissue Cell. 2024;90:102510. 39126833 10.1016/j.tice.2024.102510

[58] Lv T, Wu X, Sun L, Hu Q, Wan Y, Wang L, et al. p53-R273H upregulates neuropilin-2 to promote cell mobility and tumor metastasis. Cell Death Dis. 2017;8:e2995–e2995. 28796261 10.1038/cddis.2017.376 PMC5596564

[59] Ben Hassen C, Gutierrez-Pajares JL, Guimaraes C, Guibon R, Pinault M, Fromont G, et al. Apolipoprotein-mediated regulation of lipid metabolism induces distinctive effects in different types of breast cancer cells. Breast Cancer Res. 2020;22:38. 32321558 10.1186/s13058-020-01276-9 PMC7178965

[60] Su WP, Chen YT, Lai WW, Lin CC, Yan JJ, Su WC. Apolipoprotein E expression promotes lung adenocarcinoma proliferation and migration and as a potential survival marker in lung cancer. Lung Cancer. 2011;71:28–33. 20430468 10.1016/j.lungcan.2010.04.009

[61] Wan S, He QY, Yang Y, Liu F, Zhang X, Guo X, et al. SPARC stabilizes ApoE to induce cholesterol-dependent invasion and Sorafenib resistance in hepatocellular carcinoma. Cancer Res. 2024;84:1872–88. 38471084 10.1158/0008-5472.CAN-23-2889

[62] Jayakar SK, Loudig O, Brandwein-Gensler M, Kim RS, Ow TJ, Ustun B, et al. Apolipoprotein E promotes invasion in oral squamous cell carcinoma. Am J Pathol. 2017;187:2259–72. 28751006 10.1016/j.ajpath.2017.06.016 PMC5762938

[63] Sullivan KD, Galbraith MD, Andrysik Z, Espinosa JM. Mechanisms of transcriptional regulation by p53. Cell Death Differ. 2018;25:133–43. 29125602 10.1038/cdd.2017.174 PMC5729533

[64] Krause K, Wasner M, Reinhard W, Haugwitz U, Lange-zu Dohna C, Mössner J, et al. The tumour suppressor protein p53 can repress transcription of cyclin B. Nucleic Acids Res. 2000;28:4410–8. 11071927 10.1093/nar/28.22.4410 PMC113869

[65] Clair SS, Giono L, Varmeh-Ziaie S, Resnick-Silverman L, Liu W-j, Padi A, et al. DNA Damage-induced downregulation of Cdc25C is mediated by p53 via two independent mechanisms: one involves direct binding to the cdc25C Promoter. Mol Cell. 2004;16:725–36. 15574328 10.1016/j.molcel.2004.11.002

[66] Fischer M, Quaas M, Nickel A, Engeland K. Indirect p53-dependent transcriptional repression of Survivin, CDC25C, and PLK1 genes requires the cyclin-dependent kinase inhibitor p21/CDKN1A and CDE/CHR promoter sites binding the DREAM complex. Oncotarget 2015;6:41402. 10.18632/oncotarget.6356 PMC4747163 26595675

[67] Ho JS, Ma W, Mao DY, Benchimol S. p53-Dependent transcriptional repression of c-myc is required for G1 cell cycle arrest. Mol Cell Biol. 2005;25:7423–31. 16107691 10.1128/MCB.25.17.7423-7431.2005 PMC1190302

[68] Li H, Zhang Y, Ströse A, Tedesco D, Gurova K, Selivanova G. Integrated high-throughput analysis identifies Sp1 as a crucial determinant of p53-mediated apoptosis. Cell Death Differ. 2014;21:1493–502. 24971482 10.1038/cdd.2014.69 PMC4131181

[69] Moon SH, Huang CH, Houlihan SL, Regunath K, Freed-Pastor WA, Morris JPT, et al. p53 represses the mevalonate pathway to mediate tumor suppression. Cell. 2019;176:564–580.e19. 30580964 10.1016/j.cell.2018.11.011 PMC6483089

[70] Hassin O, Nataraj NB, Shreberk-Shaked M, Aylon Y, Yaeger R, Fontemaggi G, et al. Different hotspot p53 mutants exert distinct phenotypes and predict outcome of colorectal cancer patients. Nat Commun. 2022;13:2800. 35589715 10.1038/s41467-022-30481-7 PMC9120190

[71] Chen J, Zhu H, Chen S, Mi H. Apolipoprotein E is a potential biomarker for predicting cancer prognosis and is correlated with immune infiltration. Onco Targets Ther. 2024;17:199–214. 38523659 10.2147/OTT.S447319 PMC10960509

[72] Skrypek N, Goossens S, De Smedt E, Vandamme N, Berx G. Epithelial-to-mesenchymal transition: epigenetic reprogramming driving cellular plasticity. Trends Genet. 2017;33:943–59. 28919019 10.1016/j.tig.2017.08.004

[73] Cerami E, Gao J, Dogrusoz U, Gross BE, Sumer SO, Aksoy BA, et al. The cBio cancer genomics portal: an open platform for exploring multidimensional cancer genomics data. Cancer Discov. 2012;2:401–4. 22588877 10.1158/2159-8290.CD-12-0095 PMC3956037

[74] van Niel G, Bergam P, Di Cicco A, Hurbain I, Lo Cicero A, Dingli F, et al. Apolipoprotein E regulates amyloid formation within endosomes of pigment cells. Cell Rep. 2015;13:43–51. 26387950 10.1016/j.celrep.2015.08.057

[75] Annor GK, Elshabassy N, Lundine D, Conde DG, Xiao G, Ellison V, et al. Oligomerization of mutant p53 R273H is not required for gain-of-function chromatin associated activities. Front Cell Dev Biol. 2021;9:772315. 34881245 10.3389/fcell.2021.772315 PMC8645790

[76] Levesque AA, Pappalardo RM, Puli P, Enzor LA, Angeles C. p53 oligomerization status as an indicator of sensitivity of p53-wildtype neuroblastomas to the combination of DNA damaging agent and Chk1 inhibitor. PLoS ONE. 2022;17:e0263463. 35143532 10.1371/journal.pone.0263463 PMC8830664

[77] Friedman PN, Chen X, Bargonetti J, Prives C. The p53 protein is an unusually shaped tetramer that binds directly to DNA. Proc Natl Acad Sci USA. 1993;90:3319–23. 8475074 10.1073/pnas.90.8.3319 PMC46291

[78] Mehta S, Algie M, Al-Jabry T, McKinney C, Kannan S, Verma CS et al. Critical role for cold shock protein YB-1 in Cytokinesis. Cancers. 2020;12:2473. 10.3390/cancers12092473 PMC7565962 32882852

[79] Al Bkhetan Z, Wang S. mgikit: demultiplexing toolkit for MGI fastq files. Bioinformatics. 2024;40:btae554. 10.1093/bioinformatics/btae554 PMC11427695 39259173

[80] Cheshire C, Rönkkö T, Patel H, Ladd D, Thiery A, Fields C, et al. nf-core/cutandrun: nf-core/cutandrun v3. 2.2 Iridium Ibis. Zenodo. 2024.

[81] Ewels PA, Peltzer A, Fillinger S, Patel H, Alneberg J, Wilm A, et al. The nf-core framework for community-curated bioinformatics pipelines. Nat Biotechnol. 2020;38:276–8. 32055031 10.1038/s41587-020-0439-x

[82] Di Tommaso P, Chatzou M, Floden EW, Barja PP, Palumbo E, Notredame C. Nextflow enables reproducible computational workflows. Nat Biotechnol. 2017;35:316–9. 28398311 10.1038/nbt.3820

[83] Quinlan AR, Hall IM. BEDTools: a flexible suite of utilities for comparing genomic features. Bioinformatics. 2010;26:841–2. 20110278 10.1093/bioinformatics/btq033 PMC2832824

[84] Heinz S, Benner C, Spann N, Bertolino E, Lin YC, Laslo P, et al. Simple combinations of lineage-determining transcription factors prime cis-regulatory elements required for macrophage and B cell identities. Mol Cell. 2010;38:576–89. 20513432 10.1016/j.molcel.2010.05.004 PMC2898526

[85] Ramírez F, Ryan DP, Grüning B, Bhardwaj V, Kilpert F, Richter AS, et al. deepTools2: a next generation web server for deep-sequencing data analysis. Nucleic Acids Res. 2016;44:W160–5. 27079975 10.1093/nar/gkw257 PMC4987876

[86] Andrysik Z, Galbraith MD, Guarnieri AL, Zaccara S, Sullivan KD, Pandey A, et al. Identification of a core TP53 transcriptional program with highly distributed tumor suppressive activity. Genome Res. 2017;27:1645–57. 28904012 10.1101/gr.220533.117 PMC5630028

[87] Fischer M. Census and evaluation of p53 target genes. Oncogene. 2017;36:3943–56. 28288132 10.1038/onc.2016.502 PMC5511239

[88] Menendez D, Nguyen TA, Freudenberg JM, Mathew VJ, Anderson CW, Jothi R, et al. Diverse stresses dramatically alter genome-wide p53 binding and transactivation landscape in human cancer cells. Nucleic Acids Res. 2013;41:7286–301. 23775793 10.1093/nar/gkt504 PMC3753631

[89] Nguyen T-AT, Grimm SA, Bushel PR, Li J, Li Y, Bennett BD, et al. Revealing a human p53 universe. Nucleic Acids Res. 2018;46:8153–67. 30107566 10.1093/nar/gky720 PMC6144829

[90] DeLano WL. Pymol: an open-source molecular graphics tool. *CCP4 Newsl*. Protein Crystallogr. 2002;40:82–92.

[91] Lee TS, Cerutti DS, Mermelstein D, Lin C, LeGrand S, Giese TJ, et al. GPU-Accelerated molecular dynamics and free energy methods in Amber18: performance enhancements and new features. J Chem Inf Model. 2018;58:2043–50. 30199633 10.1021/acs.jcim.8b00462 PMC6226240

[92] Jorgensen WL, Chandrasekhar J, Madura JD, Impey RW, Klein ML. Comparison of simple potential functions for simulating liquid water. J Chem Phys. 1983;79:926–35.

[93] Maier JA, Martinez C, Kasavajhala K, Wickstrom L, Hauser KE, Simmerling C. ff14SB: improving the accuracy of protein side chain and backbone parameters from ff99SB. J Chem Theory Comput. 2015;11:3696–713. 26574453 10.1021/acs.jctc.5b00255 PMC4821407

[94] Darden T, York D, Pedersen L. Particle mesh Ewald: an N log (N) method for Ewald sums in large systems. J Chem Phys. 1993;98:10089–10089.

[95] Miyamoto S, Kollman PA. Settle: An analytical version of the SHAKE and RATTLE algorithm for rigid water models. J Comput Chem. 1992;13:952–62.

[96] Pierce LCT, Salomon-Ferrer R, Augusto F. de Oliveira C, McCammon JA, Walker RC. Routine access to millisecond time scale events with accelerated molecular dynamics. J Chem Theory Comput. 2012;8:2997–3002. 22984356 10.1021/ct300284c PMC3438784

[97] Humphrey W, Dalke A, Schulten K. VMD: visual molecular dynamics. J Mol Graph. 1996;14:33–38. 8744570 10.1016/0263-7855(96)00018-5

[98] Chew YC, Adhikary G, Wilson GM, Xu W, Eckert RL. Sulforaphane induction of p21(Cip1) cyclin-dependent kinase inhibitor expression requires p53 and Sp1 transcription factors and is p53-dependent. J Biol Chem. 2012;287:16168–78. 22427654 10.1074/jbc.M111.305292 PMC3351300

[99] Laptenko O, Beckerman R, Freulich E, Prives C. p53 binding to nucleosomes within the p21 promoter in vivo leads to nucleosome loss and transcriptional activation. Proc Natl Acad Sci USA. 2011;108:10385–90. 21606339 10.1073/pnas.1105680108 PMC3127872

[100] Dobin A, Davis CA, Schlesinger F, Drenkow J, Zaleski C, Jha S, et al. STAR: ultrafast universal RNA-seq aligner. Bioinformatics. 2013;29:15–21. 23104886 10.1093/bioinformatics/bts635 PMC3530905

[101] Anders S, Pyl PT, Huber W. HTSeq-a Python framework to work with high-throughput sequencing data. Bioinformatics. 2015;31:166–9. 25260700 10.1093/bioinformatics/btu638 PMC4287950

[102] Love MI, Huber W, Anders S. Moderated estimation of fold change and dispersion for RNA-seq data with DESeq2. Genome Biol. 2014;15:550. 25516281 10.1186/s13059-014-0550-8 PMC4302049

[103] Kuleshov MV, Jones MR, Rouillard AD, Fernandez NF, Duan Q, Wang Z, et al. Enrichr: a comprehensive gene set enrichment analysis web server 2016 update. Nucleic Acids Res. 2016;44:W90–7. 27141961 10.1093/nar/gkw377 PMC4987924

[104] Oliveros JC. Venny. An Interactive Tool for Comparing Lists with Venn’s Diagrams. http://bioinfogp.cnb.csic.es/tools/venny/index.html. 2007.

[105] Babicki S, Arndt D, Marcu A, Liang Y, Grant JR, Maciejewski A, et al. Heatmapper: web-enabled heat mapping for all. Nucleic Acids Res. 2016;44:W147–53. 27190236 10.1093/nar/gkw419 PMC4987948

[106] Adomshick V, Pu Y, Veiga-Lopez A. Automated lipid droplet quantification system for phenotypic analysis of adipocytes using CellProfiler. Toxicol Mech Methods. 2020;30:378–87. 32208812 10.1080/15376516.2020.1747124 PMC7533149

