# Supplementary material for: TP53 mutation at codon 179 metabolically reprograms cancer cells to promote invasion
Source: Cancer Gene Ther. 2026 Jun 5;33(7):775–94. doi: 10.1038/s41417-026-01045-4 (PMC13461375; doi:10.1038/s41417-026-01045-4)
Supplement: Supplementary file 1 [file 41417_2026_1045_MOESM10_ESM.docx]

**Supplementary Figure Legends**

**Supplementary Figure 1. A.** Type of *TP53* mutations reported in cancer. Data obtained from cBioPortal^73^ for the pan-cancer datasets from TCGA and MSK-IMPACT. **B.** Frequency of *TP53* mutations by codons. Data obtained from pan-cancer TCGA from cBioPortal^73^. **C.** Kaplan–Meier plots showing disease-free survival (DFS) of patients from matched tumour types in the TCGA pan-cancer dataset (via cBioPortal), stratified by tumours harbouring *TP53* mutations at R158, H193, S241, R249, P278, or R280 versus hotspot *TP53* mutations at codons 175, 220, 245, 248, 273, and 282. Sample sizes and number of events and log rank test p value for each comparison are: R158 (n=28, events=7) vs hotspot (n=272 events=70), p=0.5; H193 (n=40, events=8) vs hotspot (n=311, events=92), p=0.498; S241 (n=23, events=5) vs hotspot (n=256, events=74), p=0.44; R249 (n=22, events=10) vs hotspot (n=193, events=60), p=0.08; R278 (n=27, events=7) vs hotspot (n=298, events=82), p = 0.61; R280 (n=24, events=5) vs hotspot (n=220, events=52), p=0.31. Statistical significance was determined using the log-rank test: *p < 0.05, **p < 0.01, ***p < 0.005, ****p < 0.0001.

**Supplementary Figure 2. A.** Sanger sequencing confirming change in the base at codon 179 of the from C>G: H179R and C>T: H179Y and at codon 175 from G>A: R175H from the pCMV-TP53^WT^ plasmid. **B.** Western blot using anti-p53 antibody (DO-1) on protein lysates from H1299 cells transiently transfected with 600ng of Vo, p53^WT^, p53^R175H^, p53^H179R^ and p53^H179Y^. Anti-histone H3 antibody was used to blot Histone-H3 as a loading control. **C.** RT-qPCR showing expression of PIG3 in H1299 cells 24 hrs post-transfection with 600ng of plasmid expressing Vo, p53^WT^, p53^R175H^, p53^H179R^ and p53^H179Y^. Significance was determined using Welch tests and p < 0.05 was considered significant. *** < 0.005. **D**. Representative images and masking strategy used to quantitate the percentage of EBFP2 expressing H1299 cells 72 hrs post transfection with 600ng of plasmid expressing either Vo, p53^WT^, p53^R175H^, p53^H179R^ and p53^H179Y^. **E.** Western blot using anti-p53 antibody (DO-1) on protein lysate from isogenic H1299 cells stably expressing Vo, p53^R175H^, p53^H179R^ and p53^H179Y^. Anti-histone H3 antibody was used to blot Histone-H3 as a loading control.

**Supplementary Figure 3. A-B.** Venny shows significantly differentially **A.** Upregulated and **B.** downregulated genes in H1299 cells stably expressing p53^H179R^ and p53^H179Y^ mutants compared to Vo. Venn diagram produced using Venny^76^. **C.** Western blot showing expression of p53 using anti-p53 antibody (DO-1) in H1299 cells transiently transfected with wtp53 and Vo plasmids for 24 hours. Anti-β Actin antibody (SP124) was used to blot β-Actin as a loading control. **D.** Representative images and quantitation of neutral lipids (left) and lipid droplets (right) in H1299 cells transiently transfected with or Vo and p53^WT^ plasmids for 24 hrs and stained for neutral lipid**.** Bars ± error bars: mean ± SD. Significance was determined using Welch’s t-test and p-value < 0.05 was considered significant. ns- non-significant. **E-F.** Western blot using anti-p53 antibody (DO-1) on protein lysates from cells stably expressing Vo, p53^H179R^ and p53^H179Y^ in SKOV3 (E) and PC-3 (F)**. G.** Western blot showing levels of p53 in H1299 p53^H179R^ and p53^H179Y^ cells treated for 96 hrs with 20nM of siCntrl and si*TP53*. Anti-β Actin antibody (SP124) was used to blot β-Actin as a loading control. **H.** Representative images for quantifying neutral lipids and lipid droplets in p53^H179R^ and p53^H179Y^ H1299 cells transiently transfected for 96 hrs with 20nM control siRNA (siCntrl) or siRNA targeted *TP53* (si*TP53*). Representative images were acquired using a 60x oil-immersion objective and for quantitation, images were acquired using 20x objective.

**Supplementary Figure 4. A.** Representative images of neutral lipids in H1299 cells expressing p53^H179R^ and p53^H179Y^ mutant protein or Vo after the cells were serum starved for 60 mins and 90 mins. **B**. Western blot showing levels of p53 in H1299 p53^H179R^ and p53^H179Y^ cells treated for 96 hrs with 20nM of siCtrl and si*TP53*. Anti-β Actin antibody (SP124) was used to blot β-Actin as a loading control. **C-D.** Representative images of neutral lipids in, **(C)** p53^H179R^ and **(D)** p53^H179Y^ H1299 cells after they were transiently transfected for 72 hrs with 20nM of siCntrl or si*TP53* following which the cells were serum starved and fixed at 60 mins and 90 mins. Representative images were acquired using a 60x oil-immersion objective and for quantitation, images were acquired using 20x objective and 60x oil-immersion objective. Neutral lipid was stained with BODIPY™ 493/503 (1:1000 green) and nuclei were stained with Hoechst 33258 (2 μg/ mL, blue).

**Supplementary Figure 5. A.** Expression of *SDC1, PDLIM1, PTPRN2, GPC1, DMTN* and *RXRA* mRNA in tumours with either deleted *TP53* (*TP53*_Deep Deletion, n = 77), H179 mutations (*TP53_H179*, n = 49) or R175H mutations (*TP53_R175H*, n = 141). Bars ± error bars: mean± SEM. Significance was determined using Kruskal-Wallis test and p value < 0.05 was considered significant. Data was obtained from the pan-cancer dataset TCGA, cBioPortal^73^. **B.** Expression of *SDC1, PDLIM1, PTPRN2* and *GPC1* mRNA measured using RT-qPCR in H1299 cells expressing either Vo or p53^H179R^ and p53^H179Y^ mutants. **C.** Expression of *APOE* mRNA measured using RT-qPCR in A549 cells treated with 10μM Nutlin-3A for 24 hrs **B-C.** Bars ± error bars: mean± SEM. Significance was determined using Welch’s, Kruskal-Wallis and Mann-Whitney t-test and p < 0.05 was considered significant. * - p < 0.05, ** - p < 0.01, *** < 0.005, **** < 0.0001. **D.** *APOE* promoter occupancy enrichment by p53^WT^ in A549 cells and p53^H179R^, p53^H179Y^ and p53^R175H^ in H1299 cells treated with either solvent control (DMSO) or 10μM of nutlin-3A. The y-axis represents the normalised read density; TSS, transcription start site.

**Supplementary Figure 6.** Nitrocellulose blots stained with Ponceau S containing concentrated media from samples of **A.** H1299, **B.** SKOV3 and **C.** PC3 cells expressing Vo, p53^H179R^ and p53^H179Y^. **D.** Representative images and quantification of intracellular APOE in H1299 cells transiently transfected with either Vo or p53^WT^ for 24 hrs. Bars ± error bars: mean ± SD. Significance was determined using Welch’s t-test and p-value < 0.05 was considered significant. **** -p value < 0.0001 and ns – non-significant. **E.** Quantification of *VIM* RNA transcript abundance based on RNA-seq read counts. Bars ± error bars: mean ± SD. Significance was determined using ordinary one-way ANOVA and p-value < 0.05 was considered significant. ns – non-significant. **F.** *VIM* promoter occupancy enrichment by p53^WT^ from A549 cells and p53^H179R^, p53^H179Y^ and p53^R175H^ from H1299 cells treated with either solvent control (DMSO) or 10μM of nutlin-3A. The y-axis represents the normalised read density; TSS, transcription start site. **G**. Representative images and quantification of Vimentin in H1299 cells transiently transfected with either Vo or p53^WT^ for 24 hrs. Bars ± error bars: mean ± SD. Significance was determined using Welch’s t-test and p-value < 0.05 was considered significant. ns – non-significant. Representative images were acquired using a 60x oil-immersion objective and for quantitation, images were acquired using 20x objective and 60x oil-immersion objective.

**Supplementary Figure 7. A.** Western blot using anti-p53 antibody (DO-1) on protein lysates from cells expressing H1299 p53^H179R^ and p53^H179Y^ mutants treated for 96 hrs with 20nM of control siRNA (siCntrl), siRNA targeting *TP53* (si*TP53*) or *APOE* (si*APOE* – 5nM) for 96 hrs. Anti-β Actin antibody (SP124) was used to blot β-Actin as a loading control. **B.** Nitrocellulose blots stained with Ponceau S containing concentrated media from samples of H1299 cells expressing p53^H179R^ and p53^H179Y^ cells which were treated with control siRNA (siCntrl – 20nM) or those targeting either *TP53* (si*TP53* – 20nM) or *APOE* (si*APOE* – 5nM) for 96 hrs to ensure equal loading of all samples. **C-D**. Representative images and quantitation of neutral lipids (left) and lipid droplets (right) in **(C)** p53^H179R^ and **(D)** p53^H179Y^ H1299 cells transiently transfected for 96 hrs with 20nM control siRNA (siCntrl) or siRNA targeted to *APOE* (siAPOE). Neutral lipid was stained with BODIPY™ 493/503 (1:1000 green) and nuclei were stained with Hoechst 33258 (2 μg/ mL, blue). Bars ± error bars: Mean ± SD (n = 3-5 fields and > 200 cells)**.** Representative images were acquired using a 60x oil-immersion objective and for quantitation, images were acquired using 20x objective and 60x oil-immersion objective**.** Significance was determined using Welch’s t-test and P < 0.05 was considered significant. * -p < 0.05, ** -p < 0.01, *** - p < 0.001, **** - p < 0.0001 and ns – non-significant.

**Supplementary Figure 8. A-C.** Western blot using anti-p53 antibody (DO-1) on protein lysate from either **A.** H1299, **B.** SKOV3 and **C.** PC3 cells stably expressing Vo, p53^H179R^ and p53^H179Y^ mutant proteins. Anti β- Actin antibody (SP124) was used to blot β- Actin as a loading control. **D-F.** Representation of the z series showing one field at 20x within a dish from the bottom (Z = 0 μm) to the top (Z = 280 μm). 2% FBS was used as a chemoattractant. Nuclei of the cells are stained with 50ng/ml of Hoechst 33258 96 hrs after seeding. **D.** H1299. **E.** SKOV3 and **F.** PC3.

**Supplementary Figure 9. A-B.** RT-qPCR showing expression of *APOE* in **A.** H1299 and **B.** SKOV3 cells expressing p53^H179R^ and p53^H179Y^ mutant proteins treated with either control siRNA (siCntrl – 20nM) or those targeting either *TP53* (si*TP53* – 20nM) or *APOE* (si*APOE* – 5nM) 96 hrs post transfection. **C-D.** Representation of the z series showing one field at 20x within a dish from the bottom (Z = 0 μm) to the top (Z = 200 μm). 2% FBS was used a s chemoattractant. Nuclei were stained with 50ng/ml of Hoechst 33258 96 hrs after seeding and 72 hrs post knockdown of p53^H179R^ and p53^H179Y^ mutants. **C.** H1299 and **D.** SKOV3.
