# Supplementary material for: TP53 mutation at codon 179 metabolically reprograms cancer cells to promote invasion
Source: Cancer Gene Ther. 2026 Jun 5;33(7):775–94. doi: 10.1038/s41417-026-01045-4 (PMC13461375; doi:10.1038/s41417-026-01045-4)
Supplement: Supplementary file 2 [file 41417_2026_1045_MOESM11_ESM.docx]

Supplementary Table S2: List of reported p53 target genes.

| *ABCA12* | *CCNK* | *FAM49A* | *LMNB1* | *PLEKHF1* | *SLC30A1* | *ZNF385A* |
| --- | --- | --- | --- | --- | --- | --- |
| *ABHD4* | *CD44* | *FAM84B* | *LOC100129550* | *PLK2* | *SLC44A5* | *ZNF488* |
| *ABTB2* | *CD82* | *FAM98C* | *LOC152225* | *PLK3* | *SLC4A11* | *ZNF561* |
| *ACER2* | *CDC27* | *FAS* | *LOC201651* | *PLLP* | *SLC9A1* | *ZNF608* |
| *ACSL6* | *CDC42SE1* | *FBXO22* | *LOC399959* | *PLTP* | *SMAD3* | *ZNF79* |
| *ACTA2* | *CDH10* | *FBXO32* | *LPHN3* | *PLXNB1* | *SNX2* |  |
| *ACYP2* | *CDH8* | *FBXW7* | *LPXN* | *PLXNB2* | *SOCS4* |  |
| *ADA* | *CDIP1* | *FCHO2* | *LRDD* | *PMAIP1* | *SP110* |  |
| *ADCK3* | *CDKN1A* | *FCHSD2* | *LRP1* | *PML* | *SPATA18* |  |
| *ADGRG1* | *CEL* | *FDXR* | *LRPAP1* | *PNPO* | *SRSF8* |  |
| *ADIRF* | *CERS5* | *FGF1* | *MAD1L1* | *POLH* | *ST6GALNAC3* |  |
| *AEN* | *CES2* | *FHL2* | *MAMDC4* | *POU3F1* | *ST6GALNAC5* |  |
| *AGAP1* | *CFLAR* | *FLRT2* | *MAST4* | *PPFIBP1* | *STAT3* |  |
| *AIFM2* | *CGB7* | *FOSL1* | *MBOAT1* | *PPM1D* | *STEAP3* |  |
| *AK3* | *CHST14* | *FOXD2* | *MCC* | *PPM1J* | *STK17A* |  |
| *AKAP9* | *CLCA2* | *FOXP1* | *MCM5* | *PPP1R3C* | *STX6* |  |
| *ALDH1A3* | *CLDN1* | *FUCA1* | *MDM2* | *PPP4R3A* | *SULF2* |  |
| *ALOX5* | *CLP1* | *GADD45A* | *MELK* | *PRDM1* | *SUSD6* |  |
| *AMOTL1* | *CMBL* | *GATS* | *METTL7B* | *PRICKLE1* | *SYTL1* |  |
| *AMZ2* | *COBLL1* | *GBE1* | *METTL8* | *PRKAB1* | *TAB3* |  |
| *AMZ2P1* | *COL13A1* | *GCC2* | *MFAP3L* | *PRKAB2* | *TAP1* |  |
| *ANKRA2* | *COL7A1* | *GDF15* | *MICALCL* | *PRKX* | *TCAIM* |  |
| *ANKRD1* | *CORO2B* | *GLRX* | *MICALL1* | *PRODH* | *TEP1* |  |
| *ANKRD30BL* | *CP110* | *GLS2* | *MID1* | *PSTPIP2* | *TET2* |  |
| *ANXA4* | *CPE* | *GNAI1* | *MKNK2* | *PTAFR* | *TEX9* |  |
| *APAF1* | *CPEB2* | *GPC1* | *MLF2* | *PTP4A1* | *TGFA* |  |
| *APBB2* | *CPEB4* | *GPHN* | *MON2* | *PTPRE* | *THSD7B* |  |
| *APOBEC3C* | *CPSF4* | *GPR137C* | *MR1* | *PTPRU* | *TIGAR* |  |
| *APOBEC3H* | *CRISPLD1* | *GPR172B* | *MRPL49* | *PVRL4* | *TLR3* |  |
| *ARC* | *CROT* | *GPR87* | *MYBPHL* | *RABGGTA* | *TM7SF3* |  |
| *ARHGAP18* | *CSF1* | *GPX1* | *MYLK* | *RAD51C* | *TMBIM1* |  |
| *ARHGEF3* | *CSGALNACT1* | *GRHL3* | *MYO6* | *RALGDS* | *TMEM100* |  |
| *ARSI* | *CSNK1G1* | *GRIN2C* | *MYOF* | *RAP2B* | *TMEM200A* |  |
| *ARVCF* | *CST5* | *GSK3B* | *NADSYN1* | *RASAL2* | *TMEM63B* |  |
| *ASCC3* | *CSTA* | *HES1* | *NAV3* | *RBM38* | *TMEM68* |  |
| *ASPA* | *CTGF* | *HES2* | *NCAPD2* | *RBPMS2* | *TMEM8B* |  |
| *ASTN2* | *CTHRC1* | *HHAT* | *NEFL* | *RETSAT* | *TNFAIP8* |  |
| *ATAD2* | *CYFIP2* | *HLA-B* | *NEGR1* | *REV3L* | *TNFRSF10A* |  |
| *ATF3* | *CYP4F3* | *HLA-C* | *NF2* | *RFTN1* | *TNFRSF10B* |  |
| *ATG4A* | *DAPK1* | *HRAS* | *NFKBIA* | *RFX3* | *TNFRSF10C* |  |
| *BAX* | *DCLK1* | *HSD17B3* | *NHLH2* | *RGL1* | *TNFRSF10D* |  |
| *BBC3* | *DCP1B* | *HSPA4L* | *NINJ1* | *RGMA* | *TP53* |  |
| *BBS2* | *DDB2* | *ICAM4* | *NKAIN4* | *RGS16* | *TP53I11* |  |
| *BCL2L1* | *DDIT4* | *ICOSLG* | *NLRP1* | *RGS20* | *TP53I3* |  |
| *BCL6* | *DDR1* | *IER5* | *NMU* | *RHOC* | *TP53INP1* |  |
| *BHLHE40* | *DGKA* | *IGDCC4* | *NOTCH1* | *RNASE7* | *TP53TG1* |  |
| *BLCAP* | *DHRS3* | *IGFBP7* | *NOX4* | *RND3* | *TP73* |  |
| *BLOC1S2* | *DNAJB2* | *IKBIP* | *NTM* | *RNF144B* | *TPD52* |  |
| *BMP7* | *DNMBP* | *IL17RB* | *NTPCR* | *RNF19B* | *TRAF4* |  |
| *BNC2* | *DOCK8* | *IL1B* | *NUPR1* | *RPS19* | *TRAK1* |  |
| *BORCS7* | *DRAM1* | *IMPA2* | *NYNRIN* | *RPS27L* | *TRIAP1* |  |
| *BTBD10* | *DUSP11* | *INPP1* | *OLFML2A* | *RRAD* | *TRIM22* |  |
| *BTG1* | *DUSP14* | *IP6K3* | *ORAI3* | *RRM2B* | *TRIM32* |  |
| *BTG2* | *DUSP5* | *ISCU* | *P3H2* | *RSPO3* | *TRIM35* |  |
| *BTG3* | *DUSP7* | *ISYNA1* | *PADI4* | *RTKN2* | *TRIM38* |  |
| *C10orf88* | *DYRK3* | *ITGA2* | *PANK1* | *S100A2* | *TRIM5* |  |
| *C12orf5* | *E2F7* | *ITGA3* | *PANK2* | *SAC3D1* | *TRIML2* |  |
| *C16orf5* | *E2F8* | *ITGA6* | *PAPLN* | *SARS* | *TRIP6* |  |
| *C17orf82* | *EBI3* | *IVL* | *PAQR7* | *SCIN* | *TSGA10* |  |
| *C17orf89* | *EDA2R* | *JAG1* | *PARD6G* | *SCN2A* | *TSKU* |  |
| *C18orf1* | *EDN2* | *KANK3* | *PCBP4* | *SCN3B* | *TSPAN10* |  |
| *C18orf55* | *EFNB1* | *KCNN4* | *PCDH7* | *SCN4B* | *TSPAN11* |  |
| *C18orf56* | *EI24* | *KIAA0247* | *PCLO* | *SCRIB* | *TYMSOS* |  |
| *C19orf82* | *EML2* | *KIF15* | *PCNA* | *SDC4* | *UNC5B* |  |
| *C1orf183* | *EMP1* | *KITLG* | *PDE2A* | *SDPR* | *UQCC1* |  |
| *C1orf57* | *ENC1* | *KLHDC7A* | *PDE4C* | *SEMA3B* | *VCAN* |  |
| *C20orf108* | *ENPP2* | *KLHL13* | *PDLIM1* | *SEMA3E* | *VDR* |  |
| *C7orf10* | *EPHA2* | *KLHL30* | *PDLIM4* | *SERPINB5* | *VWCE* |  |
| *C9orf169* | *EPHA5* | *KRT15* | *PERP* | *SERPINE1* | *WDR63* |  |
| *CA12* | *EPN3* | *KRT8* | *PGAP1* | *SERTAD1* | *XPC* |  |
| *CAPN2* | *EPS8L2* | *KSR1* | *PGF* | *SESN1* | *YPEL3* |  |
| *CASP6* | *ETV7* | *LACC1* | *PGPEP1* | *SESN2* | *ZAK* |  |
| *CATSPERG* | *FAM13C* | *LAPTM5* | *PHACTR3* | *SFN* | *ZBTB5* |  |
| *CAV1* | *FAM196A* | *LASS5* | *PHLDA3* | *SLC12A4* | *ZMAT3* |  |
| *CCDC51* | *FAM198B* | *LCE1E* | *PHPT1* | *SLC16A10* | *ZNF195* |  |
| *CCDC90B* | *FAM19A2* | *LIF* | *PIDD1* | *SLC1A3* | *ZNF219* |  |
| *CCND2* | *FAM210B* | *LIMK2* | *PLCL2* | *SLC25A45* | *ZNF337* |  |
| *CCNG1* | *FAM212B* | *LMNA* | *PLCXD2* | *SLC2A12* | *ZNF367* |  |
