# Supplementary material for: TP53 mutation at codon 179 metabolically reprograms cancer cells to promote invasion
Source: Cancer Gene Ther. 2026 Jun 5;33(7):775–94. doi: 10.1038/s41417-026-01045-4 (PMC13461375; doi:10.1038/s41417-026-01045-4)
Supplement: Supplementary file 3 [file 41417_2026_1045_MOESM12_ESM.docx]

**Supplementary Table S2**: Primer details and sequences

| **Target** | **Forward Primer** | **Reverse Primer** |
| --- | --- | --- |
| *ACTB* | 5’-CCAACCGCGAGAAGATGA-3’ | 5’-CCAGAGGCGTACAGGGATAG-3’ |
| *UBC* | 5’- GCAAAGATCCAAGATAAGGAA-3 | 5’-GGACCAAGTGCAGAGTGGAC-3’ |
| *TP53* | 5’-AGGCCTTGGAACTCAAGGAT-3’ | 5’-CCCTTTTTGGACTTCAGGTG-3’ |
| *CDKN1A* | 5’-TGGAGACTCTCAGGGTCGAAA-3 | 5’-GGCGTTTGGAGTGGTAGAAATC-3’ |
| *MDM2* | 5’- GAATCATCGGACTCAGGTACATC-3 | 5’-TCTGTCTCACTAATTGCTCTCCT-3’ |
| *APOE* | 5’-CTCAGCTCCCAGGTCACCCAG-3’ | 5’-GGGTCAGTTGTTCCTCCAGT-3’ |
| *GPC1* | 5’-CACCCAGATCAGGAACCAGG-3’ | 5’-GGAAGGGACCTAGGCATTCC-3’ |
| *PTRPN2* | 5’-TGTGTGGGGTTCAGCGTAGAA-3’ | 5’-TTGGCATCATTCTGTCCGCT-3’ |
| *SDC1* | 5’-TCCAGGGGTTTCTCGCATAGG-3’ | 5’-TAGGTCCAAAGCAGTCGGATC-3’ |
| *DMTN* | 5’-TGCCTCTCTCTTCTGATGGCC-3’ | 5’-TCACCCTATTTCCACCCCACC-3’ |
| *PDLIM1* | 5’-TAAAGTGGCTGCGTCGATTG-3’ | 5’-GTTTCAGGTTGGTGCCACAG-3’ |
| *CDKN1A_Promoter ^1^* | 5’- AGCAGGCTGTGGCTCTGATT-3’ | 5’- CAAAATAGCCACCAGCCTCTTCT-3’ |
| *MDM2_Promoter ^1^* | 5’- TCAAGTTCAGACACGTTCCGAA-3’ | 5’- CTGGGAAAATGCATGGTTTAAATA-3’ |

1	Laptenko, O., Beckerman, R., Freulich, E. & Prives, C. p53 binding to nucleosomes within the p21 promoter in vivo leads to nucleosome loss and transcriptional activation. *Proc Natl Acad Sci U S A* **108**, 10385–10390 (2011). <https://doi.org/10.1073/pnas.1105680108>
